# A novel multiscale feature enhancement network using learnable density map for red clustered pepper yield estimation

**DOI:** 10.3389/fpls.2025.1548035

**Published:** 2025-04-07

**Authors:** Chenming Cheng, Jin Lei, Zicui Zhu, Lijian Lu, Zhi Wang, Jiali Tao, Xinyan Qin

**Affiliations:** ^1^ College of Mechanical and Electrical Engineering, Shihezi University, Shihezi, China; ^2^ Engineering Research Center for Production Mechanization of Oasis Characteristic Cash Crop, Ministry of Education, Shihezi, China; ^3^ Town Construction and Management Centre, 170th Regiment, 9th Division, Xinjiang Production and Construction Corps, Emin, China; ^4^ Key Laboratory of Northwest Agricultural Equipment, Ministry of Agriculture and Rural Affairs, Shihezi, China

**Keywords:** red clustered pepper, yield estimation, density map generation, Swin Transformer, hybrid dilation convolution, multiscale feature enhancement network

## Abstract

**Introduction:**

Accurate and automated yield estimation for red cluster pepper (RCP) is essential to optimise field management and resource allocation. Traditional object detection-based methods for yield estimation often suffer from time-consuming and labour-intensive annotation processes, as well as suboptimal accuracy in dense environments. To address these challenges, this paper proposes a novel multiscale feature enhancement network (MFEN) that integrates a learnable density map (LDM) for accurate RCP yield estimation.

**Methods:**

The proposed method mainly involves three key steps. First, the kernel-based density map (KDM) method was improved by integrating the Swin Transformer (ST), resulting in LDM method, which produces higher quality density maps. Then, a novel MFEN was developed to improve feature extraction from these density maps. This network combines dilation convolution, residual structures, and an attention mechanism to effectively extract features. Finally, the LDM and the MFEN were jointly trained to estimate both yield and density maps for RCP.

**Results and discussion:**

The model achieved superior accuracy in RCP yield estimation by using LDM in conjunction with MFEN for joint training. Firstly, the integration of LDM significantly improved the accuracy of the model, with a 0.98% improvement over the previous iteration. Compared to other feature extraction networks, MFEN had the lowest mean absolute error (MAE) of 5.42, root mean square error (RMSE) of 10.37 and symmetric mean absolute percentage error (SMAPE) of 11.64%. It also achieved the highest R-squared (R²) value of 0.9802 on the test dataset, beating the best performing DSNet by 0.98%. Notably, despite its multi-column structure, the model has a significant advantage in terms of parameters, with only 13.08M parameters (a reduction of 3.18M compared to the classic single-column network CSRNet). This highlights the model’s ability to achieve the highest accuracy while maintaining efficient deployment capabilities. The proposed method provides an robust algorithmic support for efficient and intelligent yield estimation in RCP.

## 1 Introduction

China has the world’s largest area under pepper cultivation. As a highly valued vegetable, pepper makes a significant contribution to the agricultural sector in terms of production value and efficiency. The growing global demand for pepper and its processed products underlines its strategic importance in Chinese agriculture. With the advancement of modern agriculture, pepper production continues to increase, necessitating the implementation of automated management practices. Automated yield estimation is crucial in this context, serving as a cornerstone in the research and development of advanced agricultural technologies. This estimation helps researchers evaluate the characteristics of different pepper varieties to improve yields, and optimises crop management strategies by monitoring the environmental impact on pepper growth. In the field of precision agriculture, yield estimation enables growers to make precise inputs tailored to the specific needs of the crop, minimising waste and maximising efficiency. However, accurate prediction of pepper yields remains a challenge, the clustered growth pattern of RCP, characterised by high fruit density and heterogeneous size distribution among individuals, combined with their predominant open-field cultivation, renders the species particularly vulnerable to environmental interference during detection processes. Therefore, the development of efficient yield estimation methods is critical.

Traditional image processing techniques have been used for plant detection and counting for automated yield estimation. However, the performance of these methods is often hampered by external factors such as lighting and background variations, which affect accurate feature extraction and reduce the accuracy and robustness of yield estimation. Apte and Patavardhan (2021) achieved quality detection of oranges and bananas by extracting colour features and texture features. Wan et al. (2022) identified and counted pineapple canopies by extracting shape, colour and texture features of pineapple canopies. Sun et al. (2019) used a dual-threshold area growth method combining colour and spatial features to extract pods and background, and proposed three geometric feature-based algorithms for pod number estimation.

Convolutional neural networks (CNNs) have developed rapidly and have made remarkable progress in various fields based on biological mechanisms of visual cognition, such as disease diagnosis, object recognition and plant segmentation (Lecun et al., 1998; Krizhevsky et al., 2017). CNNs have also been extensively applied to plant counting for yield estimation, using three main methods: detection-based counting (DC), direct regression counting (DRC), and regression density map counting (RDMC). The DC methods (Zhang et al., 2020; Hu et al., 2022) use object detection networks to identify targets within input images and generate bounding boxes for each target to determine the final count. Numerous detection models have been proposed, including Faster R-CNN (Ren et al., 2017), FPN (Lin et al., 2017), YOLO and its variants (Redmon et al., 2016; Redmon and Farhadi, 2018; Bochkovskiy et al., 2020; Li et al., 2024). For example, Bargoti and Underwood (2017) used Faster R-CNN to detect apples, almonds and mangoes. Wang et al. (2022) developed a lightweight detection model YOLOv5s-CFL for Xiaomila. Nan et al. (2023) optimised YOLOv5l for green pepper detection using a pruning algorithm based on NSGA-II. Xie et al. (2024a) improved YOLOv5s and obtained the YOLO-Ginseng model for the detection of ginseng fruits in a natural environment. However, these methods are primarily effective for non-dense, close-up targets with smaller detection areas. For dense tasks, especially those involving occlusion and overlapping targets, the annotation process is labour intensive and difficult to ensure quality, resulting in low detection accuracy and poor performance. The DRC methods (Alkhudaydi and de la Iglesia, 2022; Zaji et al., 2021) estimate quantities by direct regression, bypassing the laborious annotation process of DC methods and requiring only point annotations, thereby significantly reducing the workload. This approach is particularly advantageous for counting in complex scenarios such as crowds, dense cells and field crops. The TasselNet model series proposed by Lu et al. (2017), Lu et al. (2022) and Xiong et al. (2019) is used to count wheat ears in fields, but is highly sensitive to image angle and susceptible to background interference, resulting in poor robustness. Furthermore, the DRC methods lack the ability to visualise the spatial distribution of target objects.

The RDMC methods (Zhang et al., 2015) use density maps as regression targets, generating intermediate representations from RGB images. The model is trained on these maps, which outputs a representation of the same dimensions as the original image. By using the density map, the regressor accurately estimates the number of targets within the image. This method enhances the learning of broader contextual features by integrating additional spatial data, thereby increasing accuracy and overcoming the limitations of direct regression counting, which cannot visualise results. Taking advantage of these benefits, this counting technique was used to yield estimation in RCP. By annotating these peppers with points, the labelling effort was significantly reduced, and the density maps generated provide valuable insights into their spatial distribution. However, robust models for counting RCP in natural environments are still in their infancy. Most current methods use point-labelled density maps as ground truth (GT) via Gaussian filtering, followed using convolutional neural networks to extract features. The MCNN proposed by Zhang et al. (2016) uses convolutional kernels of different sizes to extract features from the same feature map and merge these features to generate a density map. However, the extracted shallow features are insufficient to capture the complexity of field scenes, thereby limiting the counting accuracy. Li et al. (2018) developed CSRNet to mitigate this, an improved convolutional neural network tailored for high-density scenes. This network improves the performance of several counting tasks through a cascaded dilated convolutional structure, although its single-column design struggles with targets of different scales. Liu et al. (2019) introduced the CANNet, a network designed to improve feature representation. However, its density estimation based on image planes struggles to correct for perspective distortion, compromising the robustness of the model in scenes with significant viewpoint variations. Gao et al. (2020) proposed the ASPDNet model, which excels in object counting in large-scale remote sensing data, but requires further refinement for non-remote sensing applications. Dai et al. (2021) developed the DSNet model, which addresses scale variations in crowd counting through dense dilated convolutional blocks and dense residual links. Although effective on several public datasets, its performance is suboptimal in sparse crowd conditions. Zand et al. (2022) introduced the MPS model, which features a multiscale architecture with multitask point-supervised crowd counting and localisation. While it performs well in dense, variable scenarios, its overall accuracy remains a limitation. Du et al. (2023) proposed the HMoDE model, which improves crowd-counting accuracy in dense scenarios by hierarchically mixing multiscale density experts and incorporating a relative local counting loss. However, its performance in sparse scenarios still needs improvement. Previous research have mainly focused on dense crowd counting, where crowd morphology is relatively uniform. This uniformity allows density maps generated by FDM to effectively capture spatial information, enabling general feature extraction networks to learn GT data. However, for wild RCP, the FDM fail to accurately represent spatial information due to significant variations in growth patterns and discrepancies between the Gaussian shape and the actual shape of the pepper. A training approach is used to generate learnable density maps to address above problem, as the enhanced spatial information of density maps is more beneficial for model training. Furthermore, to address the technical limitations of existing counting models, which predominantly concentrate on crowd counting scenarios and exhibit parameter excessive counts this, the study proposes a lightweight, high-precision MFEN. Designed to overcome the challenges associated with multiscale RCP fruit detection in field conditions, MFEN incorporates an innovative three-column, five-layer cascaded architecture. This architecture enables differentiated configuration of convolution kernel sizes within the triplet lightweight enhancement module group. By adhering to the principle of hybrid dilated convolution, the network dynamically adjusts dilation coefficients both within and across modules, thereby effectively suppressing grid artefacts while constructing multi-scale a feature pyramid. Consequently, the proposed framework achieves synergistic optimisation of RCP feature accuracy characterisation and model computational efficiency.

Within the of realm precision agriculture, RCP yield estimation based on vision technology encounters dual technical challenges: the paucity of publicly available annotated datasets and the absence of robust yield estimation algorithms capable of handling complex environmental conditions. To address these challenges and enhance the practical applicability of the technology, this study employs a systems engineering approach to achieve the deep integration of technological innovation and agricultural practice: Firstly, we developed a novel RCP dataset tailored for clustered crops using a multi-rotor unmanned aerial vehicle (UAV) platform. This dataset spans three fields characterised by varying crop densities and comprises 1,200 high-resolution images. Through the establishment of a comprehensive multi-task annotation framework encompassing plant localisation, disease identification, and cluster fruit counting, this dataset significantly enhances field monitoring efficiency, thereby optimising crop yield estimation. This approach aligns with the strategic of FAO direction toward sustainable intensification in agriculture. Secondly, we propose MFEN, leveraging lightweight deep metric (LDM) learning. Designed with an embedded lightweight architecture, MFEN achieves real-time processing at 130.32 FPS on experimental devices. Experimental results demonstrate that MFEN achieves an average counting accuracy of 98.02% under field conditions. Furthermore, the yield prediction model developed using MFEN significantly enhances crop planning efficiency and minimises yield losses resulting from inaccurate predictions. This network is structured with a backbone network, a multiscale enhancement module, and a density map regression module. The operational process of the network is as follows: First, annotated point information is fed into the density map generation (DMG) network for feature extraction, resulting in a density feature map. Next, the backbone network extracts shallow semantic information from the RGB image and feeds it to the feature enhancement module of the MFEN network to derive deep semantic information at different scales. The final enhanced feature maps, combined with the feature maps generated by the DMG network, are used to compute the loss, facilitating continuous optimisation of both the DMG and the MFEN. Finally, the regression module estimates both quantitative and density maps. The main contributions of this study are:

1. A high quality dataset of images of the RCP taken in natural environments at different scales has been generated. This dataset is designed to support tasks such as localisation, identification and counting of clustered peppers.
2. The KDM generation network is improved by integrating the ST, recognising the critical role of density maps in training. This modification reconfigures the feature extraction network and leverages the global modelling capabilities of the ST to improve the quality of DMG.
3. A MFEN is developed based on the multi-column structure of the MCNN. By integrating dilated convolution, residual concatenation, and an attention mechanism, the ability model of the model to extract and fuse deep contextual information has been improved without increasing the number of parameters. This improvement results in improved accuracy and stability. Evaluations on our dataset show that MFEN achieves the lowest MAE, RMSE and SMAPE values of 5.42, 10.37 and 11.64% respectively, along with the highest R² value of 0.9802. Compared to the existing methods, our model shows superior accuracy in detecting targets in dense scenes and achieves higher counting accuracy.

This paper is organised as follows: Section 2 describes the data acquisition and processing, the DMG method, and the MFEN. Section 3 discusses the training of feature extraction networks using different DMG methods and verifies the effectiveness of our approach through experiments. Section 4 provides a discussion, and the final section presents the conclusions.

## 2 Materials and methods

### 2.1 Data acquisition and data process

**Figure 1:**
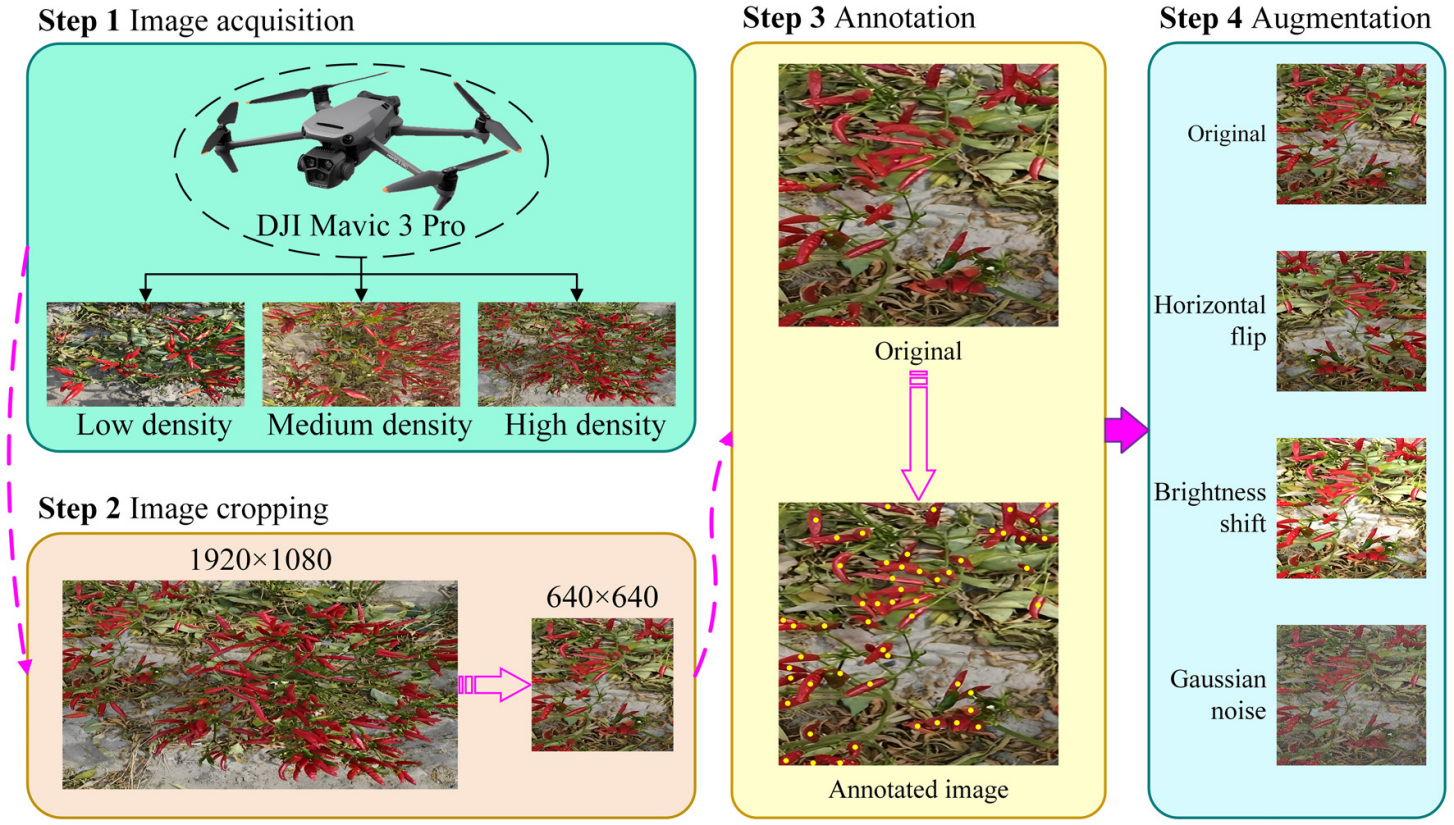
The flowchart from data acquisition to data processing.

Wild field RCP was collected to ensure the authenticity and feasibility of the data, specifically the variety Pepper Yan 908, in Hongfang Village, Cliphezi Township, Wusu City, Tacheng District, Xinjiang Uygur Autonomous Region. Data collection was conducted using a DJI Mavic 3 Pro UAV at an altitude of 2.5 metres at a slow flight speed (0.2 m/s), as shown in Step 1 of **Figure 1**. In this study, a spatio-temporal stratified sampling strategy is implemented to systematically acquire image data from experimental plots characterised by low, medium, and high growth density gradients during two critical time windows: the peak of daily light radiation (11:00-13:00) and the inflection point of diurnal temperature variation (16:00-18:00). This approach enabled the construction of a remote agricultural sensing comprehensive dataset that capture sensitively spatial the heterogeneity of biomass distribution the, multi-scale characteristics of the target objects, and the environmental variables of thermal conditions and light. Images were taken in each field by adjusting the magnification of the drone lens. The pepper samples from each field are shown in Step 1 of **Figure 1**. Prior to mechanical harvesting, attention was focused on mature, clustered peppers that were ready for harvest after defoliation. The dataset is divided into training, validation and test sets to ensure a uniform distribution of photographs at each scales, with 1,000 sample images for training, 100 for validation and 100 for testing. All images were uniformly cropped from 1920 × 1080 to a resolution of 640 × 640 in Step 2 of **Figure 1**. The composition of the dataset is detailed in **Table 1**. Data augmentation techniques are applied to increase the data richness, such as horizontal flipping, luminance transformation, and Gaussian noise addition. These methods are applied randomly during training to increase the diversity of the raw data in Step 4 of **Figure 1**.

**Table 1:** Composition and division of the datasets.

Class	Number of targets in each image				All
	13-68	69-124	125-180	181-235	
Train	250	250	250	250	1000
Val	25	25	25	25	100
Test	25	25	25	25	100
All	300	300	300	300	1200

In many conditions, targets are often small and heavily occluded, posing a challenge for box annotation. Point annotation is used to reduce the complexity of annotation and increase the efficiency of subsequent algorithmic processes. The dataset was annotated using the Labelme software, where each pepper was marked with a point at its centre. The coordinates of all the peppers within each image were recorded as their GT labels. The resulting JSON file encapsulates the 2D coordinate information for each instance. The labelled visualisation is shown in Step 3 of **Figure 1**.

### 2.2 LDM generation network

**Figure 2:**
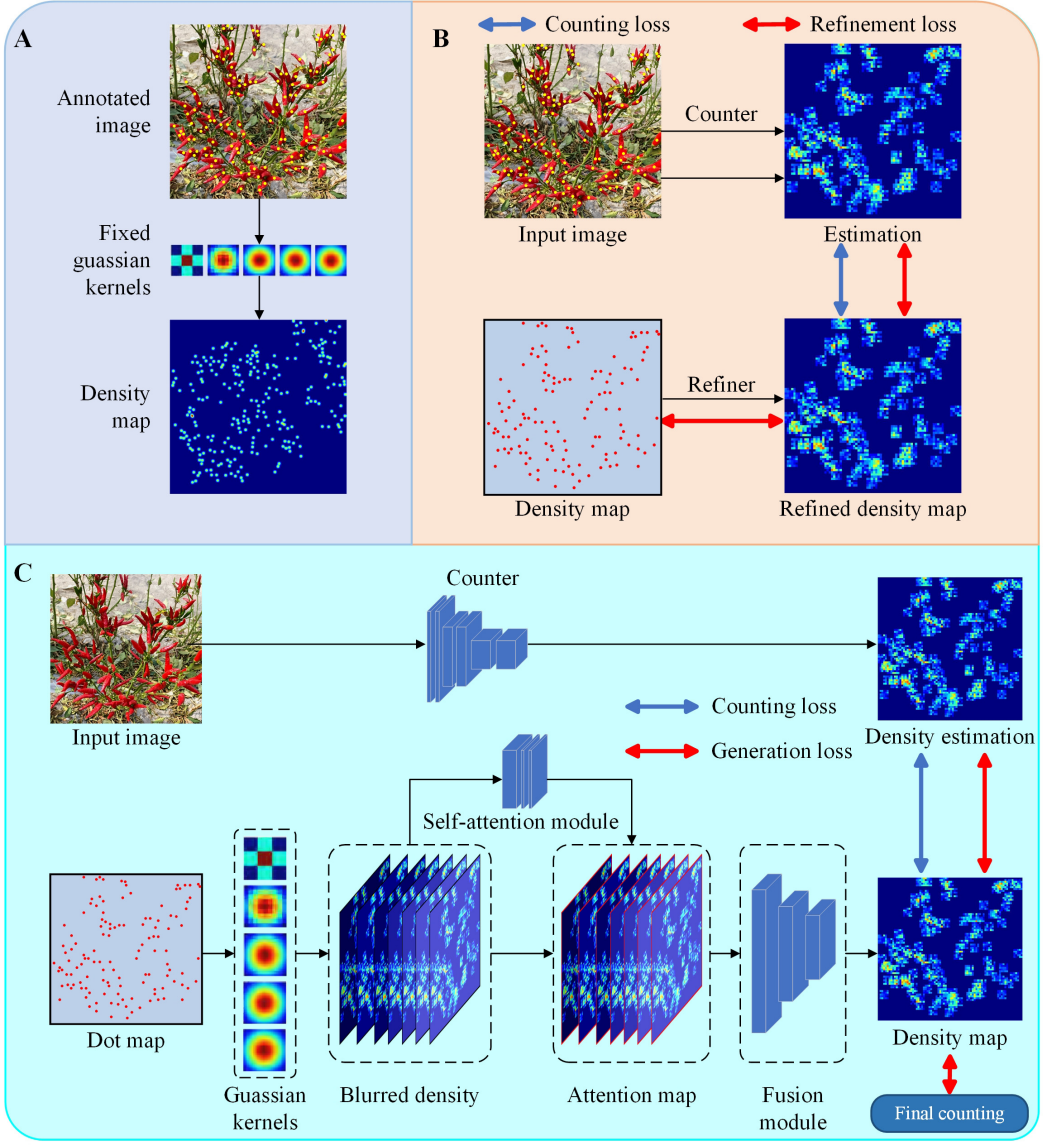
Visualisation process for constructing GT density maps of a fixed kernel density map method. **(A)** Dot map of an original image, **(B)** Target points are processed as bars with a height of 1, **(C)** Gaussian kernel convolution smoothing target points.

Direct integral estimation is a significant challenge. Regression density maps are used to reduce complexity and increase accuracy. Three common methods for generating these maps are:

1. Fixed kernel density map (FDM): This approach, converts discrete data points into a continuous soil density distribution using Gaussian kernel smoothing in **Figure 2**.
2. Refined density map (RDM): This method improves the quality of density maps produced by the fixed Gaussian kernel approach in **Figure 2**.
3. Adaptive kernel density map (ADM): This technique adaptively fuses together traditional density maps with different kernel bandwidths in **Figure 2**.

The goal of generating density maps is to simulate objects of interest across multiple poses and viewpoints, thereby maximising their spatial information and improving estimation accuracy. However, these Gaussian kernel-based approaches have certain limitations:

- The total number of peppers in the generated density maps may differ from the actual number due to potential summation bias introduced by the fusion network.
- Gaussian kernels are inadequate for targets with significantly different shapes, especially for objects such as peppers.
- Analysing individual kernel shapes is challenging because the density maps are generated directly.

A KDM method is used to produce high quality density maps for model training (Wan et al., 2022). This method effectively addresses the challenges the above challenges. Specifically, a density kernel is constructed for each object, which is then used to generate a density map centred on the object’s position. The kernel is normalised to sum to one, ensuring that the total number of objects in the density map matches the actual number of objects. In addition, the kernel shapes are explicitly defined for each object, facilitating analysis and allowing adaptability to different object types. This approach allows the framework to generate accurate density maps for RCP.

**Figure 3:**
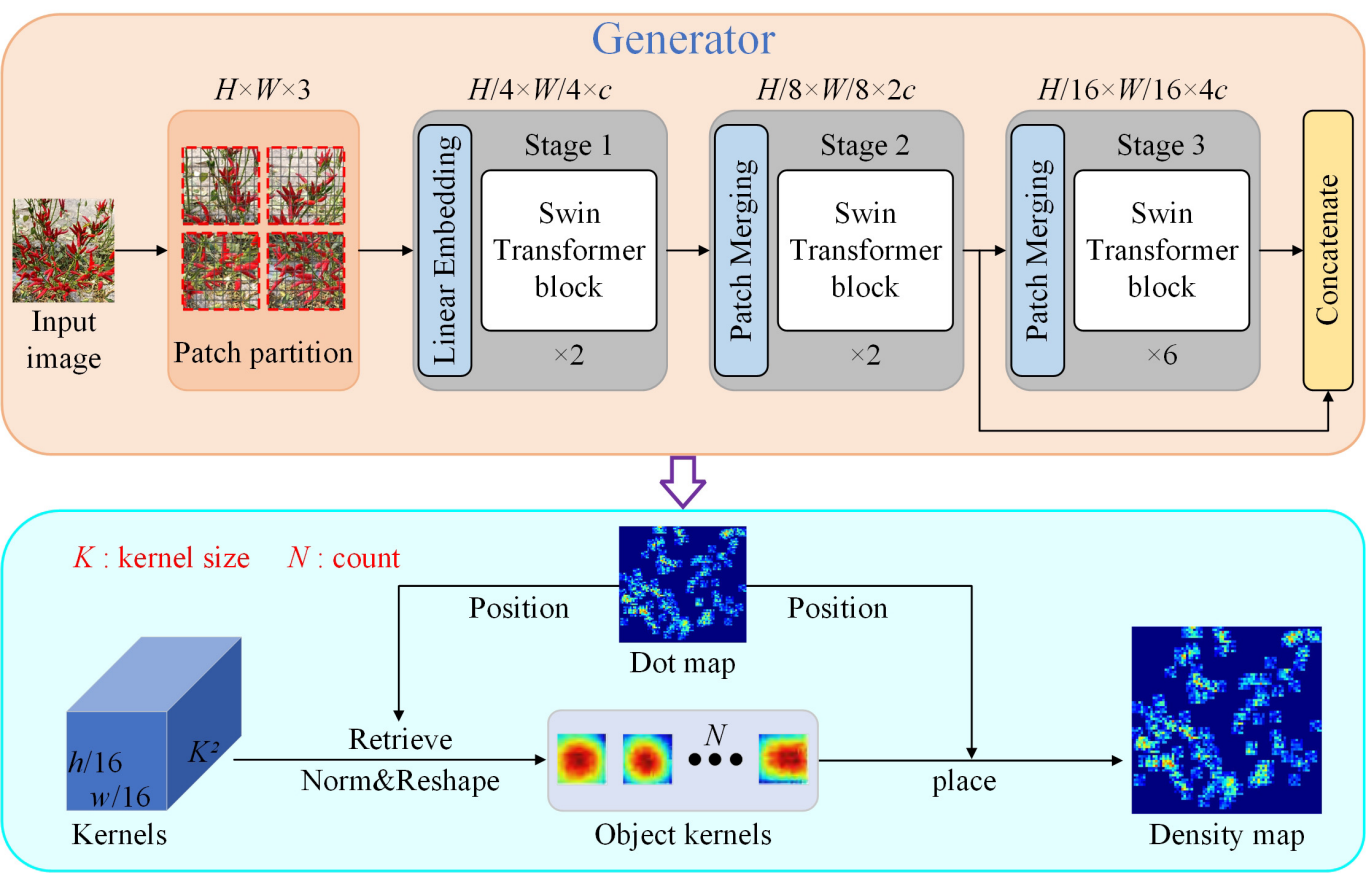
The framework for the LDM method.

In this study, the KDM method was improved, as shown in **Figure 3**. The generator module was redesigned by integrating the ST (Liu et al., 2021) as the kernel generator. The ST encoder is particularly suited to serve as a generator due to its global feature extraction capability, which is more conducive to the back end of DMG model for generating high quality density maps (Xie et al., 2024b).

**Figure 4:**
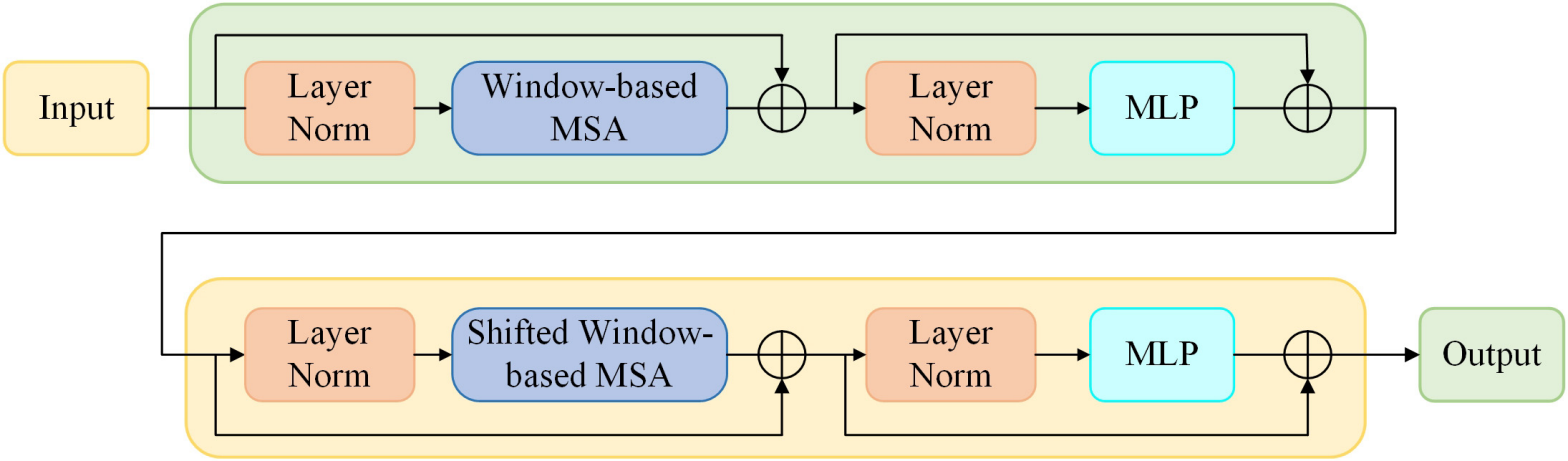
The layer structure of a ST block.

The ST has the global modelling capability of the transformer and effectively reduces the computational complexity of the transformer through the sliding window mechanism. The layered structure of the ST is shown in **Figure 4**, which includes layer normalisation, residual connections, sliding window-based multi-head self-attention (SW-MSA) and multi-layer perceptron (MLP) modules. Unlike the standard Transformer, the ST uses SW-MSA to restricts self-attention to the local window, reducing the computational complexity from $ο(P^{2})$ to $ο(PM^{2})$ , where *P* is the number of patches and *M* is the window size. For M≪P , the computational complexity of the ST is significantly lower than that of the standard global self-attention transformer. The redesigned generator architecture is illustrated in **Figure 3**. For an input image, it is divided into 4 × 4 non-overlapping patches, which are then flattened and transformed into c-dimensional features. These features are processed by the ST through a cascade of three groups. The output of the second ST block is integrated with the input of the third block to further improve performance. As a result, the final output feature map is 1/8 the size of the input image in the generator shown in **Figure 3**.

Following the LDM method process, on receiving the input image, the generator learns the kernel size associated with the spatial location of each object. It then generates a kernel map *K* of dimensions $w\times h\times k^{2}$ , where w×h is the image size and *k* is the kernel size. The kernel map K is calculated as follows:


(1) $$K=F_{k}(X)$$

where, $F_{k}$ is the generator constructed using the ST. *K* is a set of kernel mappings for the predicted output of the generator. For a given location p=(x,y) in space, the vector $K_{p}$ in *K* is a $k^{2}$ vector representing a k×k kernel. Given $\text{ℜ}={\left\{p_{j}\right\}}_{j=1}^{N}$ is a collection of two-dimensional coordinates of object annotations in an image *Y*, the corresponding kernel $K_{p_{j}}$ was retrieved from *K* for each annotation in ℜ . Then, $K_{p_{j}}$ is transformed and normalised to sum to 1, obtaining the location-specific kernel ${\tilde{K}}_{p_{j}}=vec^{-1}(K_{p_{j}})/sum(K_{p_{j}})$ of size *k* × *k*, where $vec^{-1}$ represents the vector-to-matrix transformation from $k^{2}$ -dim vector to k×k matrix and sum(ζ) is the sum of all elements in the vector ζ . Finally, the density map for training is generated by placing the generated kernel over each labelled point, calculated as follows,


(2) $$M(p)=\sum_{j=1}^{N}{\tilde{K}}_{p_{j}}(p-p_{j})$$

where ${\tilde{K}}_{p_{j}}(p)$ is indexed on p∈{−r,···,r}×{−r,···,r} , where r=(k−1)/2 , and M(p) is the final GT density map used for training.

### 2.3 MFEN

**Figure 5:**
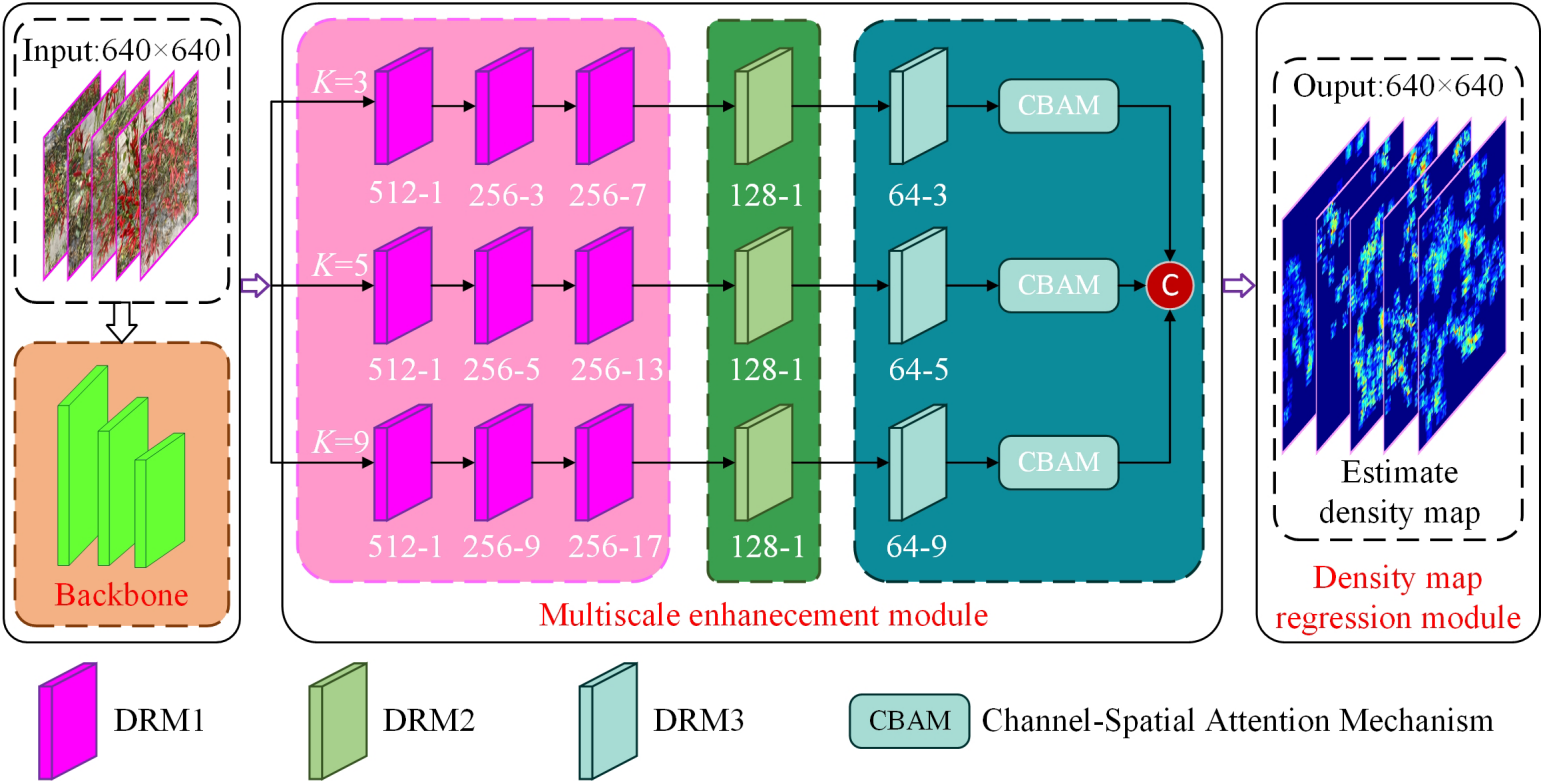
The structure of MFEN.

Outdoor images often contain background clutter and have significant scale variations in target objects. As a result, using filters with a uniform receptive field size is inadequate for capturing the features of peppers at different scales. A more effective approach is to use filters with different receptive field sizes to learn the mapping from raw pixels to density maps. Based on the successful application of Multi-column Convolutional Neural Network (MCNN) (Zhang et al., 2016), a multi-column network structure with a different size for each of the columns, combined with hybrid dilation convolution, was developed to process multiscale information. It is crucial to obtain deeper semantic information to improve feature extraction at different scales. A common strategy is to increase network depth by stacking convolutional layers, thereby improving feature extraction capabilities. However, indiscriminately increasing network depth can lead to model degradation and increased complexity. A fully convolutional dilation residual structure was designed to replace traditional convolutional layers for feature extraction to mitigate these problems, as shown in **Figure 5**. This design expands the receptive field of the model and improves accuracy. It also reduces the number of parameters and improves model stability.

**Figure 6:**
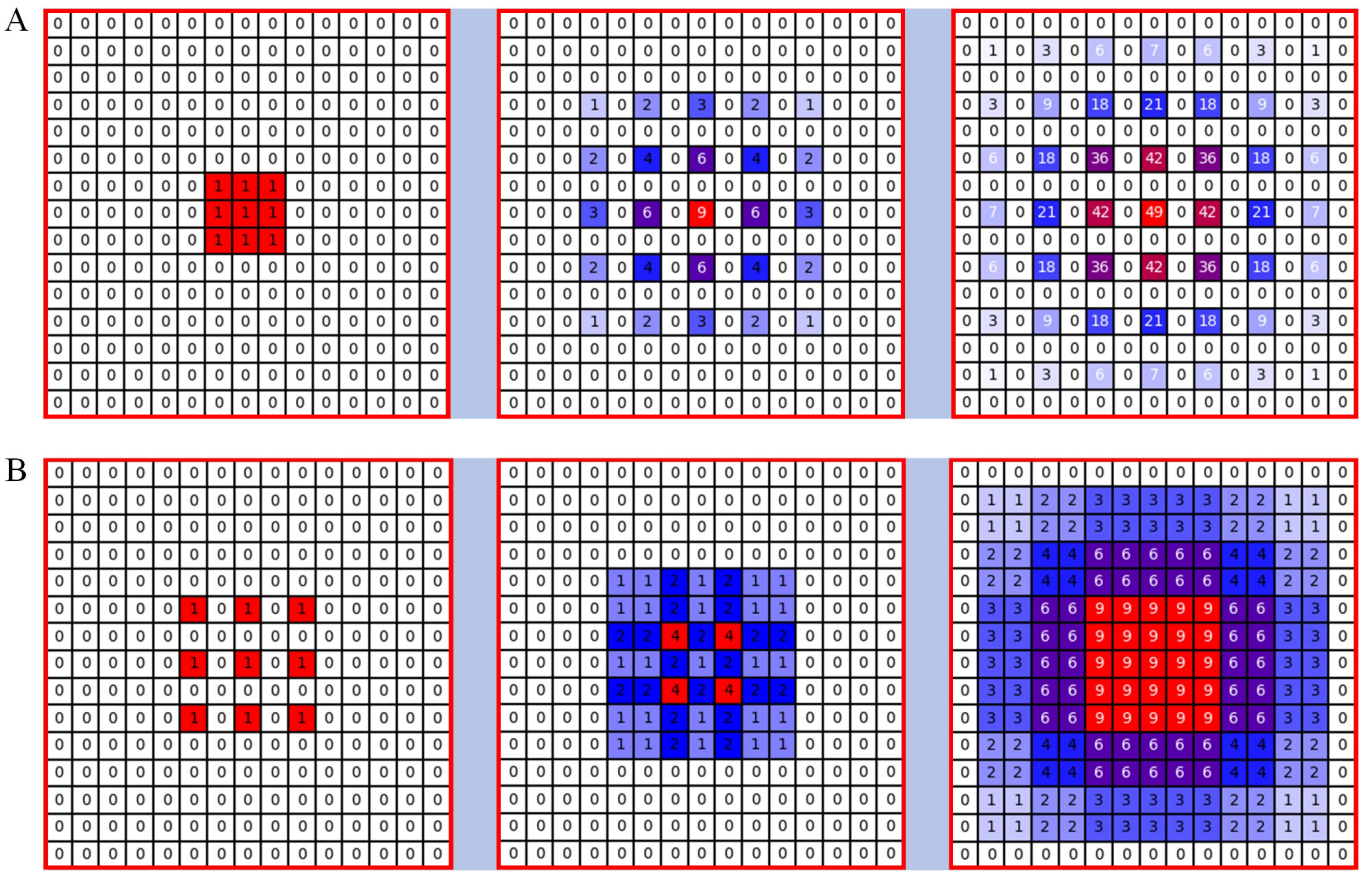
Illustration of the gridding problem. From left to right: the pixels (marked in blue) contribute to the calculation of the centre pixel (marked in red) through three convolution layers with a kernel size of 3 × 3. **(A)** All convolutional layers have a dilation rate of r = 2, **(B)** The dilated rates of the three consecutive convolutional layers are r = 1, 2 and 3 respectively.

When modelled estimated, reducing the size of the feature map can lead to the loss of pixel-level information, affecting the accuracy of the count. Pooling operations that downsample feature maps result in the loss of pixel-level semantic information by reducing resolution. Although increasing the kernel size of convolutional layers may seem like a simple solution, according to the effective receptive field (ERF) theory (Luo et al., 2016), the ERF size is proportional to the kernel size and increases linearly with depth. As a result, larger kernels significantly increase computational complexity, making it difficult to increase model depth. Dilated convolution offers an alternative, as it provides a larger receptive field compared to standard convolution in semantic segmentation tasks. For example, the CSRNet designed by Li et al. (2018) uses six layers of dilated convolutions with a dilation rate of 2 in its back-end for feature extraction, which is effective for general counting tasks. However, a theoretical problem known as the grid effect exists in the dilated convolutions of the CSRNet in **Figure 6**. Because the dilated convolution introduces zeros into the convolution kernel through the use of dilated convolution, the actual pixels that contribute to the computation from the $k_{d}\times k_{d}$ region are limited to k×k , with a gap of r−1 between them. For example, when k=3 and r=2 , only 9 out of 25 pixels are used (**Figure 6**).

Hybrid dilation convolution (HDC) (Wang et al., 2018) can be a good solution to the grid effect problem. Different layers use different dilation convolution rates, mimicking a sawtooth heuristic. Specifically, layers are grouped to form rising edges characterised by progressively increasing dilation rates, and this pattern is repeated for subsequent groups. For example, starting with the first modular layer, three successive layers are assigned dilation rates of 1, 2 and 3 respectively, and this sequence is repeated for each subsequent set of three layers. This approach allows the top layer to capture a wider range of pixel information within the same region compared to the original configuration in **Figure 6**. However, it is crucial to note that the dilation rates within a group should not have a common factor (e.g. 2, 4, 8, etc.), as this would still result in the meshing problem affecting the top layer. This distinction is fundamental to the HDC method as opposed to the atrous spatial pyramid pooling (ASPP) module (Zeiler and Fergus, 2014).

#### 2.3.1 Backbone

The first 10 layers of VGG16 (Simonyan and Zisserman, 2015) were used as the front end of our model. Leveraging its robust transfer learning capabilities and adaptable architecture, VGG16 effectively interfaces with the back-end for feature extraction. The model integrates multiple convolutional and pooling layers to construct a single-column front-end network with robust generalisation properties. For an input image In , the feature map produced by the front end is expressed as follows,


(3) $$F_{0}=F_{vgg}(In)$$

#### 2.3.2 Multiscale feature enhancement module

**Figure 7:**
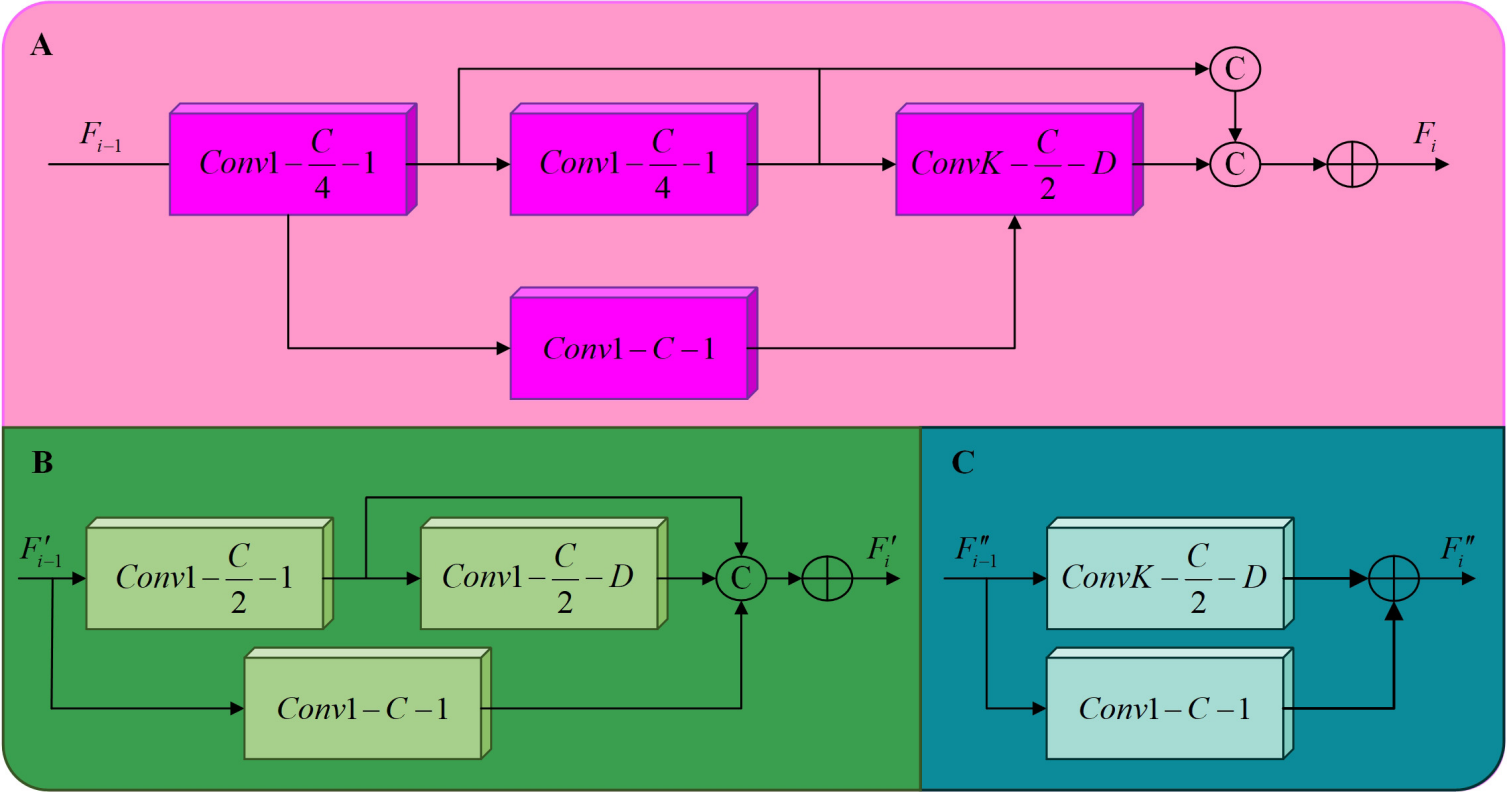
The structure of the three different dilated residual modules. **(A)** DRM1, **(B)** DRM2, **(C)** DRM3.


Three different types of dilated residual modules were designed in **Figure 7**, referred to as DRM1, DRM2, and DRM3. The DRM1 module consists of three 1 × 1 standard convolutions and a *k* × *k* dilated convolution. The DRM2 module consists of two 1 × 1 standard convolutions and a *k* × *k* dilated convolution. The DRM3 consists of a single 1 × 1 standard convolution and a *k* × *k* dilated convolution, where k∈(3,5,9) . This design has two advantages. First, it can detect objects at different scales by employing convolution kernels of different sizes. Second, it enlarges the model’s receptive field by using dilated convolutions. In the multiscale enhancement model, each column consists of a cascade of dilated residual modules, using convolution kernels of different sizes. The receptive field size of the cascaded dilated convolutions is calculated as follows,

(4) $$r_{n}=r_{n-1}+(R_{n}-1)\prod \frac{n-1}{n=1}s_{i}$$

where $r_{n}$ is the size of the receptive field in the *n*-th layer, and $R_{n}$ is the actual coverage size of the convolution kernel in the *n*-th layer, calculated as shown in Equation 5. The stride size of the *i*-th layer is denoted as $S_{i}$ . In our model, all stride sizes are set to 1. The dilation rates for the convolutions are selected as the most effective set: 3, 5, and 9. **Table 2** provides the receptive field sizes of the DRM1, DRM2, and DRM3 cascade convolutions concerning the input feature map $F_{0}$ from the front end,

**Table 2:** Theoretical receiving field for different combinations of convolution kernels and dilation rates.

Column	Kernel size	Dilation of different layers					Receptive field of different layers				
		1	2	3	4	5	1	2	3	4	5
First column	3	1	3	7	1	3	3	9	23	25	31
Second column	5	1	5	13	1	5	5	25	77	81	101
Third column	9	1	9	17	1	9	9	81	217	255	297


(5) $$R_{n}=K+(K+1)(D-1)$$

where *K* is the size of the receptive field within the dilated convolution, and *D* is the dilation rate of the dilated convolution.

In the proposed model, a CBAM is cascaded behind the DRM3 in each column, finally, the features in each column that have been refined by the CBAM are concatenated and the final feature map is generated by a convolutional layer. The architecture includes three dilated residual modules and the CBAM attention mechanism, as described below:

When a feature map of width C is input to DRM1, it first passes through two cascaded 1 × 1 convolutional layers, resulting in a reduction of the feature map size to C/4 . Then, a *k* × *k* dilated convolution is applied, where k∈(3,5,9) . The 1 × 1 convolutions improve the network’s ability to reuse information, while the *k* × *k* dilated convolution expands the model’s receptive field. This approach significantly reduces the computational complexity and the number of parameters compared to direct cascading of convolutions. The architecture of DRM1 is shown in **Figure 7**. This design effectively mitigates the problems of gradient vanishing and network degradation by incorporating features from the first two convolutional layers, thus ensuring a seamless flow of information through the back-end network. For the input feature map from the previous layer, the output of the *i*-th DRM1 is calculated as follows,


(6) $$F_{i}=(Conv_{K,1}^{\frac{C}{2}}(Conv_{1,1}^{\frac{C}{4}}(Conv_{1,1}^{\frac{C}{4}}(F_{i-1})))©(Conv_{1,1}^{\frac{C}{4}}(F_{i-1})©Conv_{1,1}^{\frac{C}{4}}(Conv_{1,1}^{\frac{C}{4}}(F_{i-1}))))⊕Conv_{1,1}^{C}(F_{i-1})$$

where $Conv_{K,D}^{C}$ is the dilated convolutional layer with an output channel *C*, kernel size *K*, and dilation rate *D*, where K∈(3,5,9) . The symbol © is the concatenation of feature maps over the channel dimension and ⊕ represents the element-wise summation of feature maps. Each column comprises three cascaded DRM1 modules, producing output feature maps $F_{3}^{1}$ , $F_{3}^{2}$ , and $F_{3}^{3}$ . Here, $F_{3}^{x}$ represents the output feature map of the third DRM1 in the *x*-th column.

Following the third DRM1 module, a cascade of a DRM2 and a DRM3 is implemented, which can reduce the number of model layers and increase the size of the feature map during training. The DRM2 module consists of two 1 × 1 standard convolutional kernels and *k* × *k* dilated convolutions, with a 1 × 1 convolution forming the residual structure, as shown in **Figure 7**. DRM3 includes a *k* × *k* dilated convolution and a 1 × 1 convolutional kernel for the residual structure in **Figure 7**. Compared to the direct cascading of DRM1, this design results in a reduction of model layers and an increase in the size of the back-end feature maps during training, improving the model’s receptive field and information extraction capability. The computation of the DRM2 output for input feature maps from the previous layer is computed as follows,


(7) $${F^{\prime }}_{i}=Conv_{K,D}^{\frac{C}{2}}(Conv_{1,1}^{\frac{C}{2}}({F^{\prime }}_{i-1}))©Conv_{1,1}^{\frac{C}{2}}({F^{\prime }}_{i-1})⊕Conv_{1,1}^{C}({F^{\prime }}_{i-1})$$

The input feature map of DRM2 is derived from the output feature map $F_{3}^{x}$ of the third DRM1. A DRM3 was cascaded after DRM2. For the input feature map ${F^{\prime \prime }}_{i-1}$ from the previous layer, the output feature map ${F^{\prime \prime }}_{i}$ from DRM3 is computed as follows,


(8) $${F^{\prime \prime }}_{i}=Conv_{K,D}^{C}({F^{\prime \prime }}_{i-1})⊕Conv_{1,1}^{C}({F^{\prime \prime }}_{i-1})$$

The input feature map for DRM3 is derived from the output feature map of DRM2.

**Figure 8:**
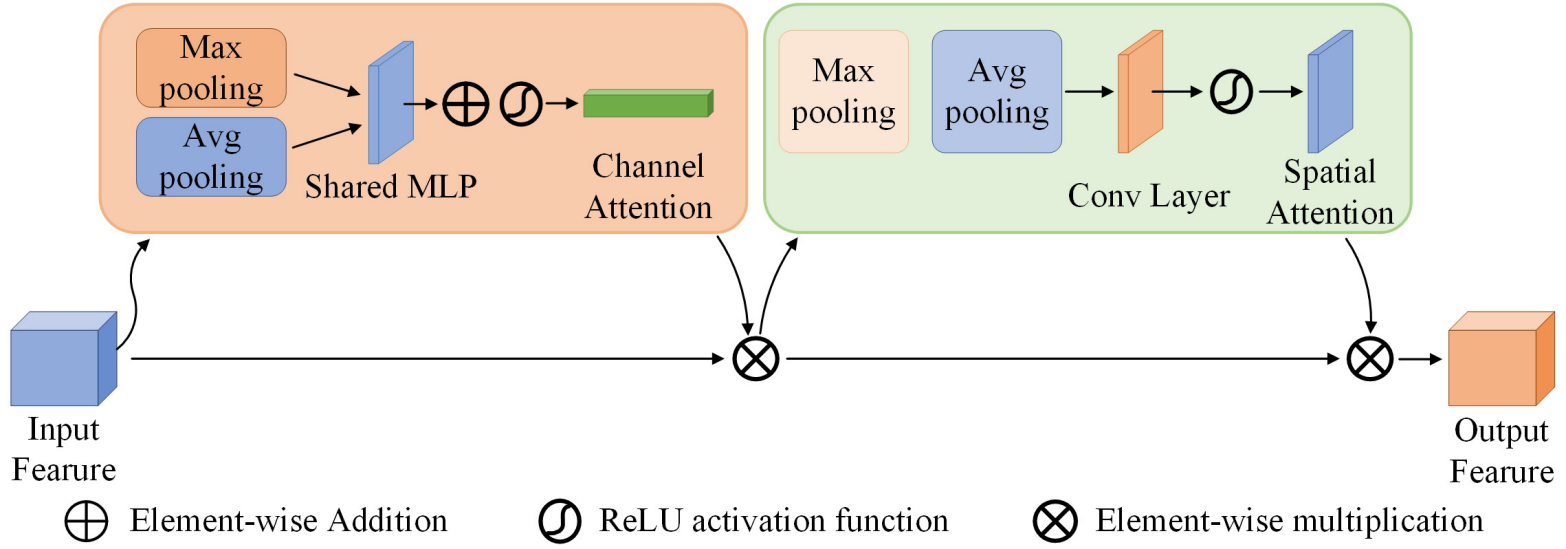
The structure of CBAM.


In neural network processing, attention mechanisms increase efficiency by selectively focusing on critical input data. Recent research has successfully integrated attention mechanisms into counting tasks to highlight important features and reduce background noise. In pepper plant detection, the overlap of stems and leaves with the background has a significant impact on accuracy. By integrating the CBAM (**Figure 8**) attention module, this problem can be mitigated (Woo et al., 2018). This module combines spatial and channel attention mechanisms to selectively enhance information-rich features and suppress irrelevant or redundant information. The spatial attention mechanism allows CBAM to adaptively adjust the weights of the feature maps, allowing the network to focus on peppers while ignoring background noise. The channel attention mechanism adaptively recalibrates the importance of different channels by capturing inter-channel dependencies, allowing the network to prioritise information-rich channels while attenuating less relevant ones. When an intermediate feature map $F\in {\mathbb{R}}^{C\times H\times W}$ is used as input, CBAM sequentially infers a one-dimensional channel attention map $M_{c}={\mathbb{R}}^{C\times 1\times 1}$ and a two-dimensional spatial attention map $M_{s}={\mathbb{R}}^{1\times H\times W}$ . The entire attention process can be summarised as follows,

(9) $$\begin{array}{l} F^{\prime }=M_{c}(F)⊗F,\\ F^{\prime \prime }=M_{s}(F^{\prime })⊗F^{\prime } \end{array}$$

where ⊗ is element-wise multiplication. During this process, the attention values are appropriately broadcast: the channel attention values are extended along the spatial dimension. The result is the final refined output $F^{\prime \prime }$ .

A CBAM is cascaded after the DRM3 in each column. The output feature map Fo is obtained by summing the CBAM output feature maps $F_{M}$ of each column, i.e.


(10) $$Fo=F_{M}^{1}⊕F_{M}^{2}⊕F_{M}^{3}$$

### 2.4 Loss function

Given a set of images and their corresponding GT point maps, the LDM method was used to generate density maps. The density map generator and the counters are trained in conjunction with our MFEN model. The loss function for this joint training is calculated as follows,


(11) $$\begin{array}{l} Loss_{1}=\sum_{i=1}^{N}\|{\hat{M}}_{i}-M_{i}\|2+\lambda (1-CS({\hat{M}}^{\prime },{M^{\prime }}_{i})),\\ CS({\hat{M}}^{\prime },{M^{\prime }}_{i})=\frac{{\hat{M}}^{\prime }\cdot M^{\prime }}{\max({\left\|{\hat{M}}^{\prime }\right\|}_{2}\cdot {\left\|M^{\prime }\right\|}_{2},\zeta )} \end{array}$$

where ${\hat{M}}_{i}$ is the estimated density map, and $M_{i}$ is the density map generated by the LDM method. The vectorised representations of ${\hat{M^{\prime }}}_{i}$ and ${M^{\prime }}_{i}$ are ${\hat{M}}_{i}$ and $M_{i}$ respectively. The cosine similarity between them is $CS({\hat{M^{\prime }}}_{i},{M^{\prime }}_{i})$ , where $\zeta =10^{-8}$ . Cosine similarity was used to spatially regularize the density maps. The normalised vectors $\hat{M_{i}^{\prime }}/{\left\|\hat{M_{i}^{\prime }}\right\|}_{2}$ and ${M^{\prime }}_{i}/{\left\|{M^{\prime }}_{i}\right\|}_{2}$ represent the spatial distributions of the populations, independent of the counts. The cosine similarity ensures that the GT and estimated density maps are more aligned in terms of spatial distribution in Equation 11.

### 2.5 Experimental settings

The network was developed using Python 3.8 on Ubuntu 20.04, with PyTorch 1.10.0 and CUDA 11.3. Both training and testing were performed on identical hardware, consisting of an Intel Xeon Silver 4214R CPU @ 2.40GHz, 90GB RAM, and an NVIDIA GeForce RTX 3080Ti GPU with 12GB VRAM. The backbone of the model is VGG16, which was initialised with pre-trained weights from ImageNet, while the remaining layers were initialised with a Gaussian distribution with a standard deviation of 0.1. In this study, a systematic grid search strategy within the Bayesian optimisation framework is used to conduct a comprehensive exploration of the hyperparameter space. Through the integration of a cross-validation mechanism (± 2.8%), we monitor in real-time the convergence trajectory of the loss function and the fluctuations in validation set accuracy. This iterative process culminates in the identification of optimal hyperparameter configurations, which include the Adam optimizer, 200 full training cycles, a batch size of 8, an initial learning rate of 7 × 10^-6^, and an L2 regularisation factor of 1 × 10^-4^,. These settings ensure stable convergence (Δloss < 1e-5/epoch) under the early stopping mechanism, thereby enhancing the robustness and reliability of the model. Basic data enhancement techniques were applied during training, including random horizontal flipping, brightness adjustment, and Gaussian noise. The GPU was preheated for an appropriate and sufficient time to ensure an accurate measurement of the inference speed.

### 2.6 Evaluation metrics

MAE, RMSE, SMAPE and R² were used as evaluation metrics. MAE quantifies the average deviation between estimated and actual values and reflects the accuracy of the model. RMSE assesses the robustness of the model, particularly in scenarios with significant errors. SMAPE measures the relative error between estimations and actuals, considering both overestimation and underestimation. As SMAPE is scale-independent, it facilitates comparisons between different models. R² assesses the fit of estimated values to actual values, helping to assess potential overfitting or underfitting. The metrics are defined as follows:


(12) $$MAE=\frac{1}{N}\sum_{i=1}^{N}|C_{i}-C_{i}^{GT}|$$

(13) $$RMSE=\sqrt{\frac{1}{N}\sum_{i=1}^{N}|C_{i}-C_{i}^{GT}{|}^{2}}$$

(14) $$SMAPE=\frac{100\%}{N}\sum_{i=1}^{N}\frac{\left|C_{i}-C_{i}^{GT}\right|}{\frac{\left|C_{i}|+|C_{i}^{GT}\right|}{2}}$$

(15) $$R^{2}=1-\frac{\sum_{i=1}^{N}|C_{i}-C_{i}^{GT}{|}^{2}}{\sum_{i=1}^{N}|C_{i}G^{T}-{\bar{C}}_{i}^{GT}{|}^{2}}$$

where *N* is the number of images in the test set, $C_{i}^{GT}$ is the true count value, and ${\bar{C}}_{i}^{GT}$ is the average count value in the test set. $C_{i}$ is the counts on the output density map whose value is the sum of the values on each pixel of the output density map.

## 3 Results

### 3.1 Comparison yield estimation accuracy with other methods

To validate the effectiveness of the proposed approach, a comparative evaluation against several state-of-the-art models was performed using on our dataset. Two DMG training methods were used: the ADM and the LDM. The models were evaluated include MCNN, CANNet, CSRNet, ASPDNet, DSNet, MPS and HMoDE. All models were trained under identical conditions and used the same data augmentation techniques.

Achieving high yield accuracy for targets of different scale, density and morphology in the presence of background clutter remains a significant challenge. **Table 3** shows that the MFEN outperforms other models in both DMG methods using the dataset. In particular, when trained using the LDM method, the MFEN has the lowest MAE, RMSE and SMAPE values of 5.42, 10.37 and 11.64% respectively, and the highest R² value of 0.9802 on the test dataset. The superior yield accuracy of the model is attributed to its multi-column structure, efficient feature augmentation, aggregation techniques, and an improved DMG method, which together increase the training effectiveness the model and significantly improve its accuracy and robustness. Although MCNN also uses a multi-column structure, its shallow network design limits effective feature extraction, resulting in a performance inferior to CSRNet, which uses a single-column structure, making it the weakest of all methods. This suggests that a multi-column structure alone is not sufficient and needs to be complemented by feature enhancement and aggregation techniques. CANNet and DSNet are the next best-performing models after MFEN, with R² values close to 0.953 and 0.97 respectively for both DMG methods. This performance is attributed to the use of dilated residual structures and aggregation techniques, which are effective in capturing contextual information. The R² value of ASPDNet under the two DMG methods are 0.9493 and 0.9656 respectively, which is slightly lower overall. Despite the use of dilated convolution and feature aggregation, the single-column structure and lack of dense residual design results in weaker contextual understanding. The R² of MPS, CSRNet and HMoDE were all less than 0.947 under the ADM method and about 0.96 under the LDM method. CSRNet, as a classic single-column model, struggles to detect targets with significant scale variation. MPS and HMoDE, both designed for dense, crowded scenes, are particularly susceptible to the DMG methods, especially when targets intersect and occlude.

**Table 3:** Comparing different methods on my dataset.

Method	Year, venue	Density map	MAE	RMSE	SMAPE	R²
MCNN	2016 CVPR	ADM	29.44	37.16	36.31%	0.6404
		LDM	27.07	34.26	34.12%	0.6838
CANNet	2018 CVPR	ADM	9.98	13.69	16.18%	0.9532
		LDM	9.05	12.71	15.28%	0.9702
CSRNet	2019 CVPR	ADM	11.61	15.67	17.87%	0.9471
		LDM	10.62	14.70	17.80%	0.9602
ASPDNet	2020 ICASSP	ADM	10.37	14.98	17.21%	0.9493
		LDM	9.60	14.66	16.81%	0.9656
DSNet	2021 ICMR	ADM	9.97	14.87	16.62%	0.9531
		LDM	9.16	12.67	16.42%	0.9704
MPS	2022 ICASSP	ADM	9.85	15.41	13.80%	0.9447
		LDM	9.25	14.78	13.01%	0.9622
HMoDE	2023 TIP	ADM	13.65	17.60	19.80%	0.9409
		LDM	12.12	15.81	17.45%	0.9604
**MFEN**	**This paper**	ADM	7.23	12.45	13.34%	0.9605
		**LDM**	**5.42**	**10.37**	**11.64%**	**0.9802**

The best results among all methods are shown in bold.

**Figure 9:**
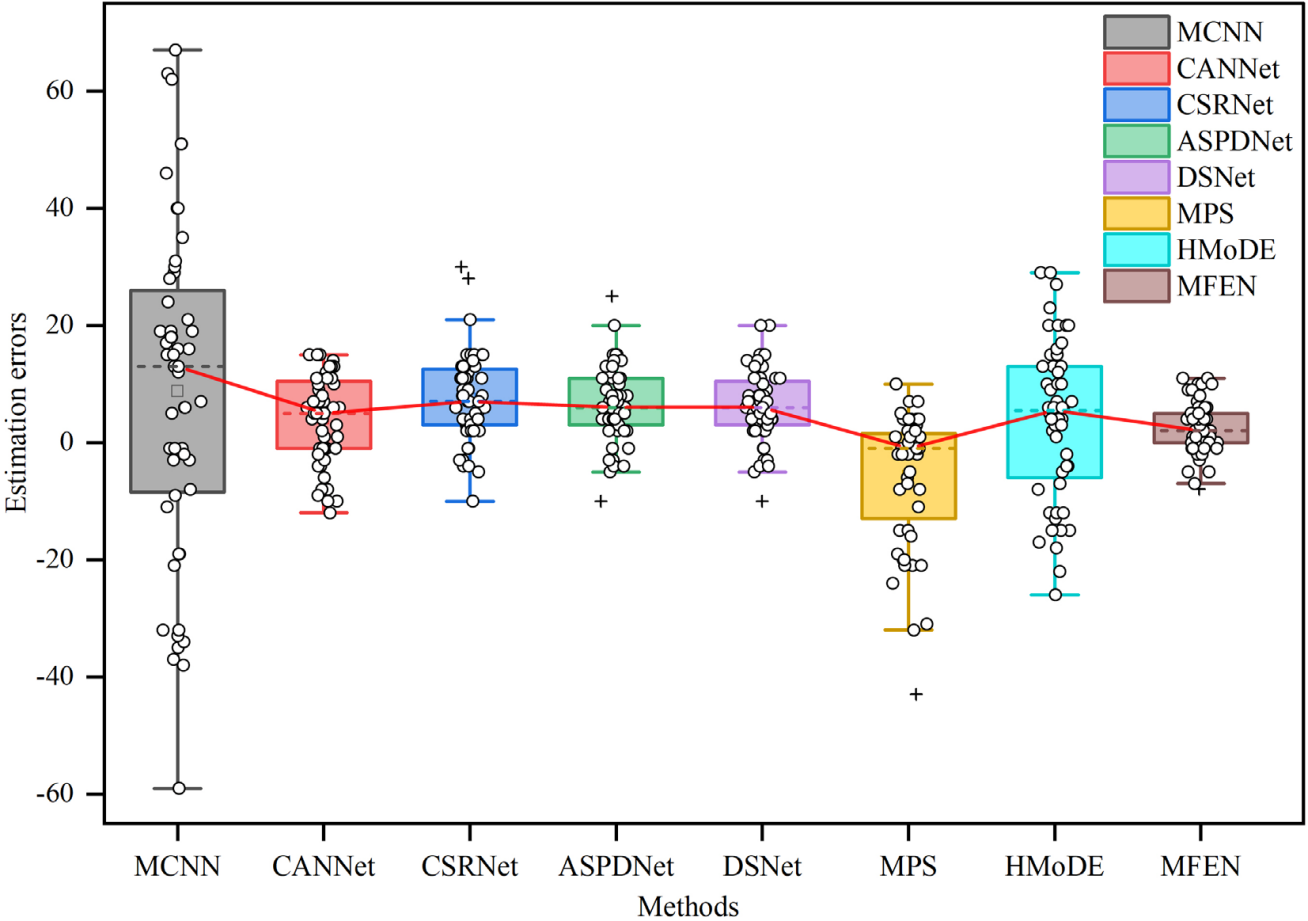
The box plots of the error values between the GT and the estimation for different methods. The upper and lower horizontal lines outside the box indicate the maximum and minimum values of the error, the top and bottom of the box indicate the upper and lower quartiles respectively, the dotted line inside the box indicates the mean value of the error, the circle indicates the individual error and the plus sign indicates the outliers.

A box plot was generated to further illustrate the discrepancy between estimated and actual yields for the different models, which were trained by the LDM method, as shown in **Figure 9**. The analysis shows that the maximum error in MCNN estimations exceeds 60, and the average error exceeds the upper quartile of other methods, highlighting the inferior accuracy and robustness of MCNN, making it unsuitable for practical applications. The data distribution shows that CANNet, CSRNet, ASPDNet, DSNet and HMoDE tend to overestimate yield, with their plots skewed towards the positive y-axis and containing some anomalies. This is mainly due to their sensitivity to background and target scale transformations, resulting in misclassification of background as targets. Nevertheless, their overall accuracy remains high, with average errors not exceeding 10. Conversely, MPS generally underestimates the estimated yield with large errors and overestimates with smaller errors, suggesting frequent missed target identifications. MFEN has the shortest box heights and the smallest absolute values for upper and lower bounds, indicating that its estimations have the most consistent data fluctuations with errors centred between zero and five and the lowest estimation errors. Although there are a few outliers, they remain within acceptable limits compared to other methods. As a result, MFEN outperforms other models in terms of both accuracy and stability of yield estimation, particularly in scenarios with dense, scale-varying targets.

### 3.2 Comparison density map estimation with other methods

**Figure 10:**
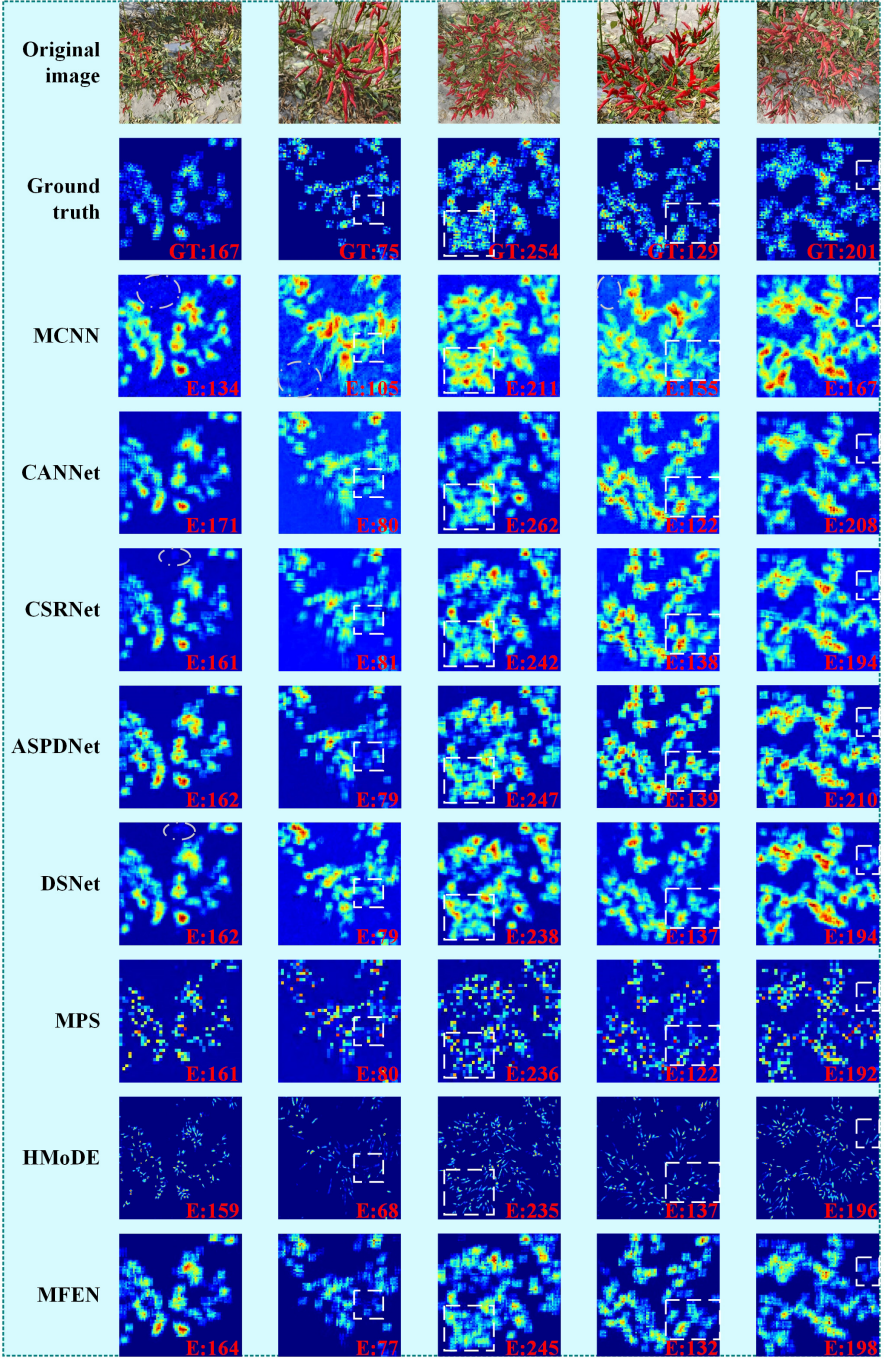
Density maps derived from my dataset using different methods. The white elliptical areas represent obvious identification stray points, and the white rectangular areas represent areas where there are obvious differences in identification.

Five images are selected for validation, with sparse small targets, sparse large targets, dense small targets, dense large targets, and a mix of dense large and small targets. **Figure 10** shows the estimated density maps and counting results of the different methods for these images, arranged from left to right. The results show that our proposed MFEN accurately locates pepper fruits and effectively discriminates the background, producing density maps closest to the GT and achieving superior yield estimation accuracy. For example, the density maps estimated by MFEN for both sparse small and large target images, especially the latter, are close to the GT, resulting in minimal counting errors (see the first and second columns). In contrast, MCNN’s density maps are significantly affected by background and pepper pose, showing numerous stray points (see the white elliptical areas) that deviate from the GT, leading to overestimation and underestimation.

Similarly, MPS and HMoDE are also affected by background noise and pose variations, resulting in missed targets on the density maps and consequently low yield estimations. The differences in performance on dense targets are shown in the third and fourth columns. In scenarios with occluded or intertwined small targets that are densely packed, the estimated density map often shows high-density values at specific locations, with lower values in the surrounding areas, resulting in underestimation. Conversely, for densely packed large targets, the density values tend to be high, resulting in an overestimation (see the rectangular areas). For example, MCNN has the largest error, over 10%, while CSRNet, DSNet, MPS and HMoDE have errors between 5% and 10%. CANNet, ASPDNet and MFEN have smaller errors, all within 5%. Overall, MFEN excels in both density map estimation and yield estimation, demonstrating robust performance across different scenarios. In the fifth image, characterised by significant target scale variation and concentration, all methods show locally high estimated density values, resulting in overestimated yields (see the rectangular areas). MCNN is the most affected, while MFEN and HMoDE are the least affected. HMoDE benefits from multiscale hierarchical density mixing and local technique loss, while the success of MFEN is attributed to its multi-column structure, feature enhancement module and aggregation techniques. Despite MFEN’s strong performance, occlusion and interweaving with the background still affect yield estimation accuracy in dense small target scenarios, resulting in locally high-density values and missing potential targets (see third column). A more efficient feature extraction architecture is used to replace VGG16, and infrared imagery is incorporated to improve accuracy in scenes with dense target interweaving and occlusion.

### 3.3 Comparison inference speed and the parameters with other methods

**Figure 11:**
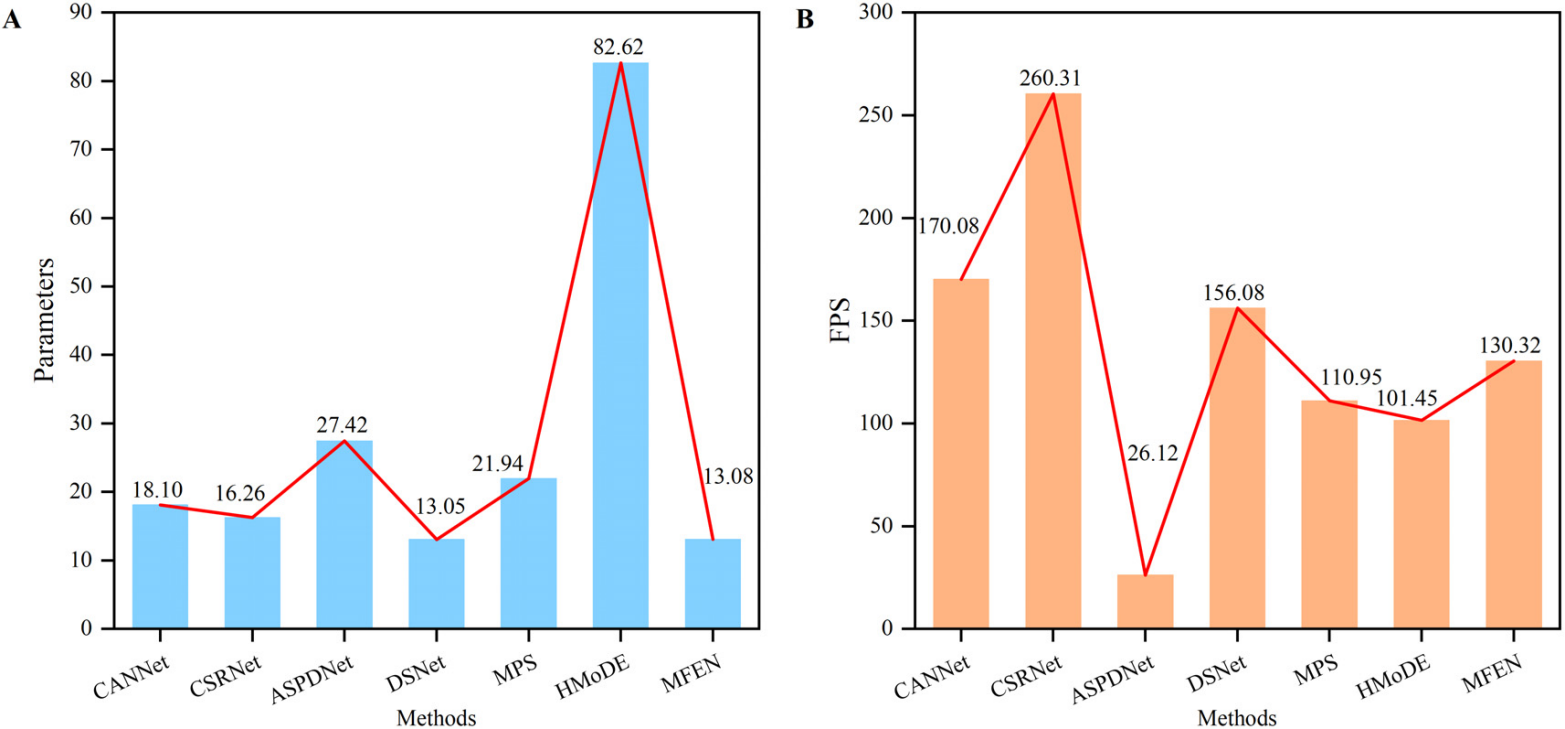
The parameters and inference speed of different methods. **(A)** The parameters of different methods, **(B)** The FPS of different methods.

Input images with a resolution of 640 × 640 were used to assess the inference speed of each model. After ensuring that the training and validation metrics had stabilised, the performance of the methods that met the accuracy threshold was evaluated (R² ≥ 0.9), as shown in **Figure 11**. Although CSRNet demonstrated a fast inference speed (260 FPS), its application to yield estimation of RCP was suboptimal, resulting in low accuracy and inaccurate density maps. This suggests that a simple single-column dilated convolutional model is inadequate for handling complex multiscale targets. Both MPS and HMoDE showed comparable accuracy to CSRNet, but their inference speed was less than half that of CSRNet, and they had significantly larger parameter counts, particularly HMoDE with 82.62M parameters, which poses challenges for deployment. CANNet and DSNet improved accuracy at a slight cost to inference speed, achieving a more balanced performance in terms of inference speed, accuracy and parameter size. However, MFEN outperformed all other models by significantly reducing parameters through the cascading of DRM1, DRM2 and DRM3, resulting in 3.16M fewer parameters than CSRNet and an R² value of over 0.98. Despite its moderate inference speed (130.32 FPS), the R^2^ value of MFEN outperformed that of all the models compared, surpassing DSNet, which was previously the best of the models compared, by 1%. To further evaluate how well MFEN’s performance aligns with real-world requirements, its inference capability was compared with the demands of real-time applications. Real-time applications typically strict impose constraints time on model inference to meet the necessity for instantaneous feedback. For instance, in scenarios such as video surveillance or industrial automation, processing speeds exceeding 100 FPS are often required. With an inference speed of 130.32 FPS, MFEN satisfies real-time requirements while achieving an R² value exceeding 0.98, demonstrating exceptional accuracy in yield estimation tasks. This combination of high accuracy and rapid inference capability ensures that MFEN performs exceptionally well in practical applications In. conclusion, MFEN offers a robust solution for RCP yield estimation scenarios diverse across, owing to its superior accuracy and efficient inference performance.

### 3.4 The impact of different DMG methods

**Figure 12:**
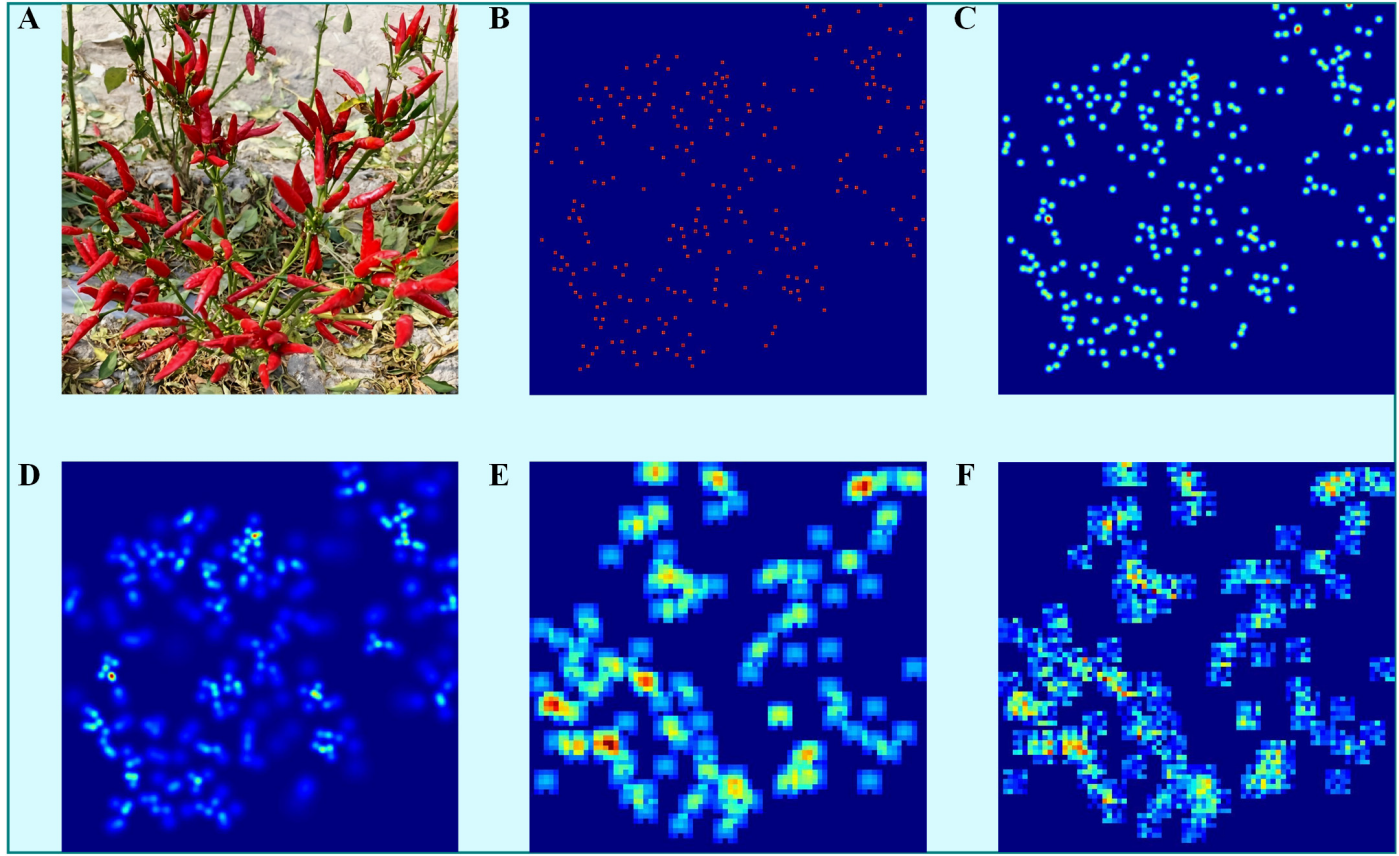
Visualisation of density maps for different DMG methods. **(A)** Original image, **(B)** FDM (kernel size = 4), **(C)** FDM (kernel size = 16), **(D)** ADM, **(E)** KDM, **(F)** LDM.

The effects of the FDM, the ADM, the KDM and the LDM methods were evaluated to assess the influence of different density map generation (DMG) techniques on model performance. **Figure 12** illustrates the GT density maps produced by these methods. The first three methods, FDM (**Figure 12**), ADM (**Figure 12**), are not training-based and are categorised as conventional DMG methods. These methods primarily use a Gaussian function to smooth the target coordinates on a 2D plane, thereby producing feature representations rich in information. For the FDM method, kernel sizes of 4 and 16 were used. The last two methods, KDM (**Figure 12**) and LDM (**Figure 12**), are training-based and aim to capture spatial position, shape and pose information to improve the generation of GT density maps for pepper fruits. MFEN and DSNet are used to evaluate the impact of the different DMG methods. The FDM method with a kernel size of 16 outperformed that with a kernel size of 4, demonstrating better suitability for small targets (**Table 4**). Among MFEN and DSNet, the models trained with the ADM method showed the lowest accuracy, with R² values of 0.9605 and 0.9531, respectively. This discrepancy is attributed to the algorithm’s automatic kernel size adjustment, which is susceptible to occlusion and interleaving, thereby misleading model learning. Both KDM and LDM outperformed the non-training-based methods, significantly improving model accuracy. For MFEN, the KDM method reduced the MAE and RMSE by 2.20 and 0.44 respectively and improved R² value by 0.93% compared to the FDM method with a kernel size of 16. The LDM method further reduced the MAE and RMSE to 1.37 and 2.40 respectively and improved R² value by 0.98%. In summary, the LDM method outperformed the others, mainly due to the global modelling capability of the ST, which accurately captures target distribution and scale variations, surpassing the capabilities of simple convolutional and pooling layers. **Figure 12** shows the density map generated by the KDM method, while **Figure 12** shows the LDM method. It is clear that the LDM method focuses more on overall target distribution and background differentiation rather than local targets, making it more effective in scenarios involving occlusion and interweaving.

**Table 4:** The impact of different density maps on model performance.

Model	Density Map	MAE	RMSE	R²
MFEN	FDM (kernel size = 16)	8.99	13.21	0.9611
	FDM (kernel size = 4)	9.78	13.63	0.9595
	ADM	7.23	12.45	0.9605
	KDM	6.79	12.27	0.9704
	**LDM (ours)**	**5.42**	**10.37**	**0.9802**
DSNet	FDM (kernel size = 16)	9.16	14.55	0.9551
	FDM (kernel size = 4)	9.88	14.89	0.9501
	ADM	9.97	14.87	0.9531
	KDM	9.68	13.54	0.9622
	**LDM (ours)**	**9.16**	**12.67**	**0.9704**

The best results of each method are shown in bold.

### 3.5 The impact of different dilated rate configurations

A comparison of three configurations is conducted, while maintaining the same network architecture, to assess the effect of dilation rates on multi-column cascade dilation convolution. The first configuration uses standard convolution. The second configuration, like CSRNet, sets the dilation rate for all dilation convolution layers to 2. The third configuration uses a hybrid dilation convolution design. The accuracy and inference speed of these configurations were evaluated at 640 × 640 resolution, using frames per second (FPS) as the metric, as detailed in **Table 5**. The results show that replacing the dilation convolution with the standard convolution (configuration 1) results in a marginal decrease in inference speed and a reduction in R² value of approximately 1.99%. Using a fixed dilation rate of 2 (configuration 2) significantly slows down the inference speed and reduces R² value by 1.16%. In contrast, our proposed hybrid dilation rate convolution configuration (configuration 3) demonstrates superior performance in both speed and accuracy. This improvement is mainly attributed to our hybrid dilation rate design, which effectively mitigates the grid effect associated with fixed dilation rates, reduces redundancy, expands the model’s receptive field, improves the understanding of multiscale targets, and achieves higher accuracy and inference speed.

**Table 5:** Estimation accuracy and speed performance of different dilation rate configurations.

Configuration	MAE	RMSE	SMAPE	R²	FPS
Configuration 1	6.82	12.07	13.79%	0.9603	125.30
Configuration 2	6.49	11.51	12.64%	0.9686	102.10
**Configuration 3 (ours)**	**5.42**	**10.37**	**11.64%**	**0.9802**	**130.32**

The best results of each configuration are shown in bold.

### 3.6 The impact of different kernel size configurations

A comparison of four different configurations is conducted, while keeping the network architecture constant, to assess the influence of different convolution kernel sizes on the performance of a multi-column cascade dilation convolution network. In the first configuration, the convolution kernel sizes for the dilation layers across all columns of the feature-enhanced network were set to 3, with the aim of minimising model size and optimising deployment efficiency. The second configuration used convolution kernel sizes of 3, 5 and 7 for the respective columns. The third configuration used kernel sizes of 3, 5 and 9, while the fourth configuration used kernel sizes of 3, 7 and 9. Configuration 1 had the smallest model parameters (9.94M) but the lowest accuracy, with a R² value of 0.9613 (**Table 6**). As the convolution kernel size increased, so did the number of model parameters, with the accuracy peaking in configuration 3, which reached a R² value of 0.9802. Further escalation of the kernel size in configuration 4 resulted in a decrease in accuracy (a 0.7% decrease in R² value compared to configuration 3) and an increase in model parameters (0.99M more than configuration 3), compromising feasibility.

**Table 6:** Estimation accuracy and inference speed of different convolutional kernel configurations.

Kernel size configuration			Evaluation metrics					
Column 1	Column 2	Column 3	Parameter	MAE	RMSE	SMAPE	R²	FPS
3	3	3	9.94M	6.70	11.31	13.65%	0.9613	125.12
3	5	7	12.01M	6.21	11.28	13.09%	0.9689	126.32
**3**	**5**	**9**	**13.08M**	**5.42**	**10.37**	**11.64%**	**0.9802**	**130.32**
3	7	9	14.07M	6.49	11.50	12.62%	0.9732	124.21

The best results of each configuration are shown in bold.

### 3.7 Benefits from dilated residual module and attention mechanism

Two validation strategies were used to assess the effectiveness of the key components in our network architecture. First, the necessity of each module was examined by evaluating different combinations of dilated residual and attention mechanism modules. Second, the role of the dilated residual module was evaluated by replacing the DRM1, DRM2, and DRM3 with standard convolutional layers of equivalent depth, but without the residual structure. Several baseline models were generated for both validation methods in **Table 7**.

**Table 7:** The baseline models established for different validation methods.

Validation method	Baseline model	Combination or replacement rules
Combination	Baseline 1	Five DRM1
	Baseline 2	Three DRM1 and two DRM2
	Baseline 3	Three DRM1 and two DRM3
	Baseline 4	Three DRM1, one DRM3 and one DRM3
Replacement	Baseline 1	DRM1, DRM2 and DRM3
	Baseline 2	DRM1 and DRM2
	Baseline 3	DRM1 and DRM3
	Baseline 4	DRM2 and DRM3
	Baseline 5	DRM1
	Baseline 6	DRM2
	Baseline 7	DRM3

**Figure 13:**
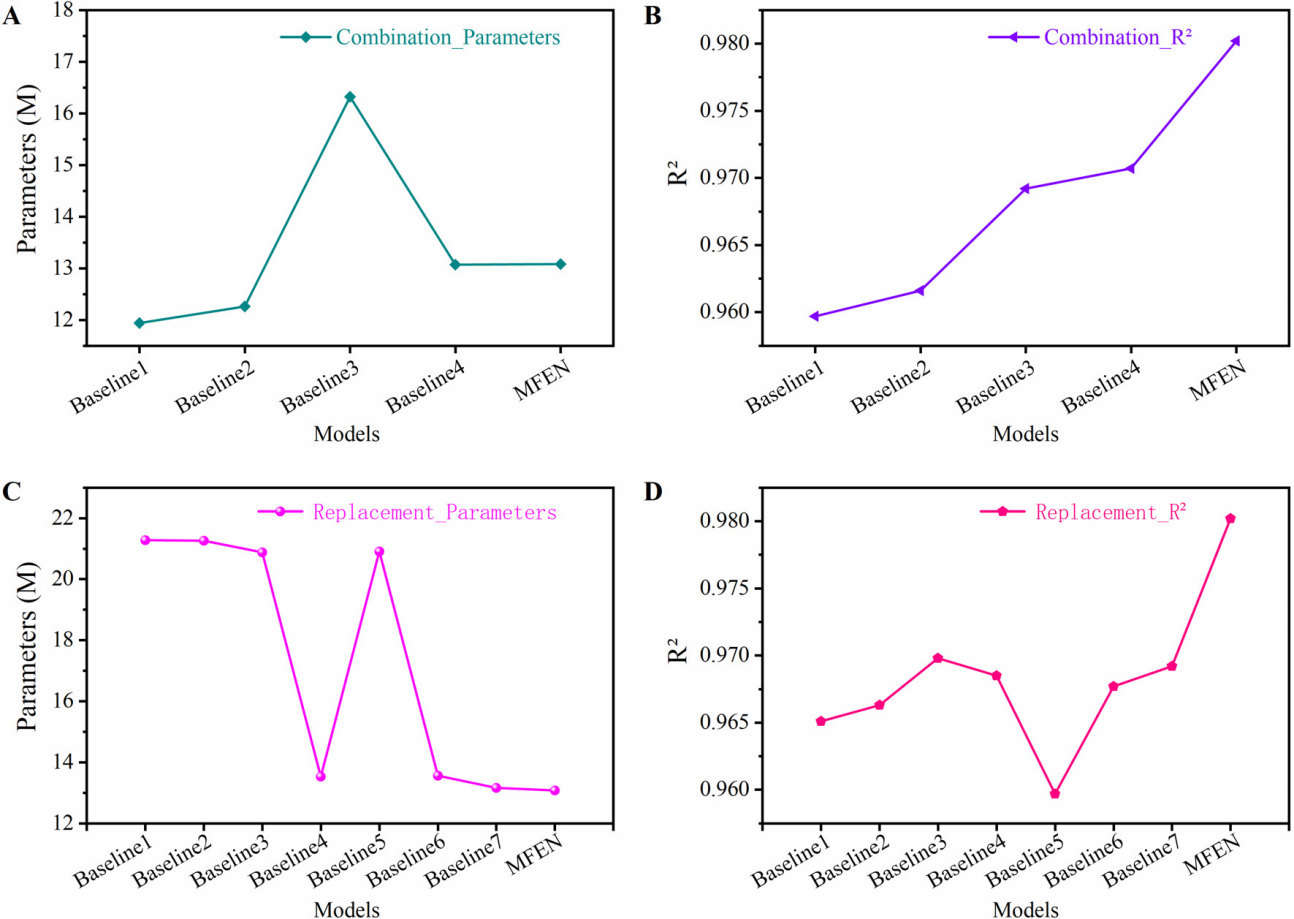
The parameters and FPS of different models. **(A)** Different models’ parameters of combination, **(B)** Different models’ R² of combination, **(C)** Different models’ parameters of replacement, **(D)** Different models’ R² of replacement.

Experiments were performed on the dataset to evaluate the performance of each baseline model, and the results are listed in **Table 8**. The analysis shows that using DRM1 and DRM2 reduces model parameters by 37.49% and 3.61% respectively and improves accuracy. Although DRM3 does not significantly reduce parameters, it significantly improves accuracy by increasing the size of the feature map at the back end of the model. In the first validation method, DRM1 uses three 1 × 1 convolutions for feature compression and aggregation, reducing the feature map to a quarter of the input size during forward propagation. This effectively reduces parameters in wider slices. However, the reduced feature map size at the front end makes DRM1 unsuitable for stacking throughout the model, resulting in the lowest accuracy (e.g. Baseline 1 in **Figure 13**). In contrast, DRM2 and DRM3 increase the feature map size at the back end by minimising the use of 1 × 1 convolutions. Stacking DRM2 and DRM3 at the back end significantly improves model accuracy (e.g. Baseline 2, Baseline 3 and Baseline 4 in **Figure 13**). However, stacking only DRM3 significantly increases the number of parameters (e.g. Baseline 3 in **Figure 13**) and its accuracy is inferior to Baseline 4, which has fewer parameters. In summary, stacking DRM1 at the front end effectively reduces parameters while maintaining accuracy, and DRM2 and DRM3 can be stacked at the back end to increase feature maps, significantly improving accuracy, albeit with some increase in parameters. The CBAM attention mechanism is also crucial; without it, the model cannot reach the upper limit of the R² value (0.9802) and only reaches 0.9707 (compare Baseline 4 in **Figure 13** with MFEN).

**Table 8:** Effects on estimation accuracy of different dilated residual modules and attention mechanisms.

Method	Model	Parameter	MAE	RMSE	SMAPE	R²
Combination	Baseline 1	11.94M	7.48	12.95	14.71%	0.9597
	Baseline 2	12.26M	6.67	12.11	13.28%	0.9616
	Baseline 3	16.32M	6.32	11.80	13.17%	0.9692
	Baseline 4	13.07M	6.31	11.67	13.01%	0.9707
Replacement	Baseline 1	21.28M	7.98	12.96	13.80%	0.9651
	Baseline 2	21.26M	8.35	12.27	14.97%	0.9663
	Baseline 3	20.88M	6.88	11.35	13.40%	0.9698
	Baseline 4	13.53M	6.92	12.20	13.63%	0.9685
	Baseline 5	20.91M	9.20	14.01	16.34%	0.9597
	Baseline 6	13.56M	7.23	12.54	14.35%	0.9677
	Baseline 7	13.16M	7.20	12.32	14.59%	0.9692
**Origin**	**MFEN (ours)**	**13.08M**	**5.42**	**10.37**	**11.64%**	**0.9802**

The best results among all models are shown in bold.

The second validation approach involves the evaluation of the dilated residual module. By substituting the three dilated residual modules and the attention mechanism module, a contrasting Baseline model was constructed. When all modules are substituted, the model has the highest number of parameters and the lower R² value, which is 0.9651 (**Figure 13**). DRM1 significantly reduces the number of model parameters while maintaining the accuracy of the model (compare **Figure 13** for Baseline 4, Baseline 5 and MFEN). In contrast, the combined use of DRM2 and DRM3 increases the size of the feature map during forward propagation of the model to the backend, resulting in an increase of 1.17% in R² (compare **Figure 13** for Baseline 4 and MFEN). This improvement is crucial for the extraction of small target features. Therefore, this validation further confirms the effectiveness of our design approach.

## 4 Discussion

### 4.1 Effect of LDM generation method

A training-based approach was used to generate GT density maps to optimize the accuracy of yield estimation. The feature extractor within the KDM framework was augmented by integrating the ST, which uses its global modelling capabilities to produce superior density maps. Our experimental evaluation compared the FDM, ADM, KDM and LDM methods, as detailed in **Table 4**. The results show that the traditional non-training-based methods (FDM and ADM) show negligible differences in estimation accuracy, with variations not exceeding 0.5%. This discrepancy is attributed to the irregularity of the targets and the randomness of their poses, which makes it difficult to accurately represent spatial relationships. In addition, the misalignment of the Gaussian kernel with the target shape contributes to suboptimal results. Using the MFEN trained with each DMG method, the R² value for traditional methods is approximately 0.96. The KDM method achieves a R² value of 0.9704, an improvement of 0.93% over the best traditional method. The LDM method achieves a R² value of 0.9802, an improvement of 0.98% over the previous KDM version, and has the lowest MAE and RMSE values of 5.42 and 10.37 respectively. Visualisation of the density maps shows that training-based methods not only localise target positions but also account for pose and shape variations (e.g. **Figures 12E**), in contrast to traditional methods (e.g. **Figures 12B**). As the feature extractor is refined, the focus of the model becomes more comprehensive. This indicates that the ST, with its global modelling capabilities, allows for better differentiation between background and targets, as well as between individual targets. Compared to the convolutional and pooling based extractor used in KDM, the ST significantly improves model accuracy.

While the LDM method demonstrates outstanding performance in experimental settings, its applicability and effectiveness are subject to certain limitations. Below, we outline the primary scenarios that may result in suboptimal outcomes for LDM methods:

- Data Dependency: The efficacy of the LDM method is heavily contingent upon the quality and diversity of the training data. If the training dataset lacks representation of specific critical scenarios—such as extreme lighting conditions, target occlusions, or regions with unusually high densities—the model may exhibit diminished performance when encountering these situations in real-world applications
- Model Generalisation Ability: The LDM approach exhibits limited adaptability to variations in target pose and shape. In complex operational environments characterised by overlapping targets or highly irregular poses, the model may struggle to deliver accurate judgments, potentially leading to misclassifications or omissions.
- Robustness: The resilience of the LDM method to noise and external interference requires further enhancement. Under extreme interference conditions, such as abrupt changes in lighting, pronounced shadows, or significant background disturbances, the model’s performance may degrade, compromising its reliability in challenging environments.

### 4.2 Effect of MFEN

A comprehensive evaluation of different model configurations tailored to our tasks was performed, including different dilation rates (**Table 5**), convolution kernels (**Table 6**), and the integration of dilation residue modules with attention mechanisms (**Table 8**). The results show that the proposed configuration, which incorporates optimised dilation rates, convolution kernels, and module combinations, achieves superior accuracy and inference speed. Specifically, our model yields MAE, RMSE, SMAPE, R², and FPS values of 5.42, 10.37, 11.64%, 0.9802, and 130.32, respectively. This configuration effectively balances model parameters, inference speed, and accuracy, prioritising accuracy while maintaining competitive inference speed and parameters efficiency to ensure robust deployment performance. MFEN achieves the fastest speed of inference and the highest level of accuracy in **Table 5**. Although MFEN does not have the smallest number of parameters or the absolute fastest speed, its accuracy significantly outperforms other configurations (**Table 6**), which is why we chose convolution kernel sizes of 3, 5 and 9. In contrast, Baseline configurations such as Baseline 1 and Baseline 2 (**Table 8**), despite their lower parameters, fail to achieve the same level of accuracy as our proposed scheme. Through rigorous experimental comparisons, the configuration that excelled in both accuracy and deployment performance was identified, leading to the development of the MFEN model.

Compared to other density map estimation and yield estimation methods, including MCNN, CANNet, CSRNet, ASPDNet, DSNet, MPS and HMoDE, MFEN shows superior performance, as evidenced by the data in **Table 3**. Under the two different DMG methods, MFEN achieves R² values of 0.9665 and 0.9802, outperforming the next-best methods by 0.83% and 0.98% respectively. In addition, MFEN has the lowest MAE, RMSE and SMAPE metrics of the alternatives evaluated. Examining the yield estimation errors and density map quality in **Figures 9**, **10**, MFEN’s error box plots have the shortest height, indicating minimal and consistent data fluctuations and the lowest counting inaccuracies. Density maps generated by MFEN closely resemble actual conditions, effectively distinguishing between background and target elements while minimising yield estimation errors. In contrast, other methods often struggle to accurately distinguish between background and targets, resulting in higher error.

It is important to note that training and deploying MFEN on less powerful hardware presents several challenges. First, the training process of MFEN requires substantial computational resources and memory support. In environments with limited resources, this can lead to significantly increased training times or even failure to complete the training task. Second, while MFEN demonstrates relatively fast inference speeds in experimental settings (130.32 FPS), it may not meet the requirements of real-time applications on certain low-performance hardware.

### 4.3 Limitations and future work

There are three main limitations to the current method. First, the operating parameters of the UAV platform are constrained by strict thresholds: at an altitude of 2.5 metres and standard optical zoom conditions, the flight speed must be controlled to within 0.5 m/s to ensure image clarity; and when the flight altitude is increased to 3.5 metres, the target resolution drops dramatically below the thresholds required for effective detection. Secondly, the static image analysis protocol faces a dynamic performance bottleneck and lacks a compensation mechanism to cope with airflow disturbances (e.g. an average wind speed of 3.2 m/s at a height of 1.5 metres) or the shading effect of a high-density canopy (6 to 10 plants per square metre). Thirdly, as the data set was collected in a fixed viewing angle mode (top view, 2.5 metres height), the generalisability of the method is limited when applied to agricultural platforms with different mounting angles and different RCP species.

To address the above shortcomings, the follow-up work will focus on the following three aspects. In terms of optimising dynamic performance, a spatio-temporal fusion framework combining optical flow motion field estimation with ConvLSTM multi-frame weighting will be established, and motion blur will be reduced by an adaptive optical zoom coordination mechanism at an airspeed not exceeding 0.7 m/s. To improve scale adaptation, a multi-height synthetic dataset at 0.3 metres intervals (covering a height range of 2.5 to 3.5 metres) will be used, and cross-domain adaptive training based on CycleGAN will be applied to ensure that the loss of scene accuracy at untrained air heights does not exceed 5%. Embedded mission optimisation will then use FPGA-accelerated lightweight networks to ensure that the inference process reaches 30 FPS while maintaining over 95% accuracy. In addition, a dynamic data acquisition protocol will be developed to create a quantitative modulation model between flight speed/altitude/optical zoom, and a transfer learning framework based on an attention mechanism will be developed to enhance the adaptability of the model to common RCP variants.

## 5 Conclusions

This paper presents an improved DMG method, termed LDM, and a novel model, MFEN, aimed at estimating RCP yields and producing high quality density maps. The main results are as follows:

- The KDM method was improved by integrating the ST module into its feature extraction network to improve the quality of the GT DMG. This integration enabled the network to capture feature information globally. Compared to the KDM method, LDM, when trained jointly with MFEN, showed an increase in R² value of 0.98% and a reduction in MAE and RMSE of 1.37 and 2.40 respectively, significantly improving the accuracy of the MFEN.
- Different dilation rate configurations, convolution kernel configurations, combinations of dilation residual modules and attention mechanism modules were evaluated to develop a yield estimation model that balances accuracy and deployment performance. This led to the configuration of the MFEN with optimal overall performance. Several models (MCNN, CANNet, CSRNet, ASPDNet, DSNet, MPS, HMoDE and MFEN) were trained using the LDM and ADM methods. The results show that MFEN outperforms the others, achieving the lowest MAE, RMSE and SMAPE metrics of 5.42, 10.37 and 11.64% respectively. It is the only model with R² value above 0.98, which is 0.98% higher than DSNet, the best-performing model in the comparison. In addition, MFEN has 13.08M parameters, 3.18M fewer than the classic single-column model CSRNet, and achieves an inference speed of 130.32 FPS on a single 640 × 640 RGB image, which meets practical requirements.

While the current work has yielded promising results, several research directions remain to be explored, offering opportunities for further advancements:

- Hyperspectral Data Integration: Integrating hyperspectral data with existing visual data could leverage the rich spectral information to enhance the model’s feature extraction capabilities and classification accuracy, particularly in complex or visually similar environments. This integration could enable the model to distinguish subtle differences in materials or objects that are challenging to discern using data visual alone.
- Dataset Expansion: Expanding the dataset’s size and diversity by collecting additional samples under varied conditions—such as different lighting scenarios, target densities, poses, and levels of background clutter—could validate the model’s adaptability across a broader range of practical applications. A more comprehensive dataset would not only improve generalisation but also help identify potential weaknesses in the current model design.
- Multimodal Data Fusion: Exploring the fusion of visual data with complementary sensor data, such as LIDAR or infrared sensors, could enhance the model’s robustness under constrained conditions. By leveraging the unique strengths of each modality, the fused system could achieve more reliable performance in challenging environments where visual data alone may be insufficient.
- Model Structure Optimisation: Further optimising the model architecture by exploring lightweight module designs or incorporating techniques such as knowledge distillation could reduce the number of model parameters and computational complexity while maintaining high accuracy. Such optimisations would make the model more deployable on resource-constrained hardware, broadening its applicability in real-world scenarios.

## Data Availability

The original contributions presented in the study are included in the article/supplementary material. Further inquiries can be directed to the corresponding author.
